# The Repeatome in the Mega-Genus *Epidendrum* L. (Epidendroideae, Orchidaceae): An In Silico Comparative Analysis

**DOI:** 10.3390/genes17020161

**Published:** 2026-01-30

**Authors:** Ana Carolina Humberto, Magdalena Vaio, Ana Paula Moraes

**Affiliations:** 1 Graduate Program in Evolution and Diversity, Federal University of ABC, Alameda da Universidade s/n, São Bernardo do Campo 09606-045, SP, Brazil; ana.humberto@ufabc.edu.br; 2 Laboratorio de Evolución y Domesticación de las Plantas, Facultad de Agronomía, Universidad de la República, Garzón 780, Montevideo 12900, Uruguay; mvaio@fagro.edu.uy; 3 Laboratory of Plant Cytogenomics and Evolution, Department of Biodiversity and Biostatistics, Institute of Biosciences, São Paulo State University—UNESP, Rua Prof. Dr. Antonio Celso Wagner Zanin 250, Botucatu 18618-689, SP, Brazil

**Keywords:** repeatome, repetitive DNA, satellite DNA, transposable elements

## Abstract

**Background/Objectives:** Variation in repeatome composition is a major determinant of genome architecture and an important substrate for evolutionary change in plants. Despite the availability of genomic sequence data, repeatome-wide assessments have not been performed for *Epidendrum*, the largest Neotropical genus of Orchidaceae. Here, we assessed repeatome profiles across 34 *Epidendrum* species using publicly available genomic datasets. **Methods:** *Epidendrum* repeatomes were characterized with the RepeatExplorer2 pipeline, and patterns of repeat composition were evaluated for phylogenetic structure using a species phylogeny. **Results:** Repeat composition showed no clear phylogenetic structure, with closely related species often displaying divergent satDNA and TE profiles. satDNA content varied widely among species (15.5–69% of the repeatome fraction). A total of 208 satDNA families were detected, which were used to build a custom database for comparative analyses. We detected 73 satDNA clusters shared among species, whereas only three were species-specific. Regarding TEs, Class I elements were the most abundant repeats, dominated by Ty3-Gypsy LTR retrotransposons. Only two Class II TIR superfamilies were detected (EnSpm/CACTA and hAT). **Conclusions:** This study provides the first comprehensive characterization of the *Epidendrum* repeatome and establishes a resource for future work on cytogenomic diversity within this megagenus. The heterogeneous distribution of repeats among closely related species is consistent with lineage-specific amplification and loss, highlighting rapid repeatome turnover in *Epidendrum*. Potential drivers, as hybridization and ecological differentiation, should be tested explicitly in future analyses integrating broader genome size sampling and trait data.

## 1. Introduction

Genome size (GS) in plants is shaped by the dynamic interaction of recurrent and shared evolutionary processes throughout their history [1]. The GS, also referred as C-value (where C refers to constant), represents the DNA content of the unreplicated haploid chromosome complement [2,3] and can vary through two mechanisms: changes in chromosome number via aneuploidy or polyploidy, and/or expansions and contractions of repetitive DNA sequences, collectively known as the repeatome [4,5,6,7]. These sequences can be classified into two major types: (1) tandemly repeated DNA sequences, which are repeated two or more times sequentially in the same genomic region, such as satellite DNA (satDNA; [8,9,10,11]), and (2) sequences dispersed along the chromosomes, the transposable elements (TEs).

In plants, satDNA may comprise from 0.1% to 50.43% of the genome, and despite the existence of numerous species-specific satDNAs, some families are widely distributed and shared across different organisms, from the genus to the phylum level [10]. According to the library hypothesis, proposed by Salser et al. (1976) [12], related taxa share a repertoire (“library”) of satDNA sequences, whose presence is largely conserved among species. Such genomic repeatome stability is suggested to favor hybridization [13]. However, genomic prominence of different satDNA is caused primarily through quantitative amplification and contraction over time. In each species, certain members of this library may be amplified, while other satellite sequences are present at low/undetectable levels. According to this model, new satDNA families in different evolutionary lineages can be explained by diversification of satDNA from the original library. The rapid amplification of a satDNA from the library can considerably alter the genomic profile of chromosomal arrangements and create reproductive barriers between organisms, promoting speciation [10,14]. By amplifying or contracting their repeats, satDNAs can contribute to GS variation among species; however, TEs are considered the main drivers of GS differences [9,10].

The TEs are divided into two main classes based on their transposition mechanism. TEs of Class I, the retrotransposons, move through a “copy-and-paste” mechanism, resulting in a greater prevalence of retrotransposons in plant genomes [15,16], while TEs of Class II (DNA transposons) move through a “cut-and-paste” mechanism [15,17]. The most common TEs in plants are retrotransposons belonging to the long terminal repeat (LTR) subclass. Taxonomically, LTR retrotransposons are classified into two main superfamilies: Ty1-Copia and Ty3-Gypsy, which are distinguished mainly by the order of their protein-coding domains and are subdivided into other major evolutionary lineages [15,18,19,20,21,22,23,24,25].

Within host genomes, TEs can proliferate through transposition bursts when they evade cellular surveillance, but deleterious insertions are normally restricted by epigenetic silencing mechanisms [16,26]. Although TE insertions may be harmful, most are neutral and some can even become adaptive under certain conditions [16]. While TEs do not directly drive speciation, shifts in their abundance after species divergence increase genomic polymorphism [18]. Such changes may trigger chromosomal rearrangements and non-homologous recombination between TE-rich regions [18,27], ultimately affecting heterochromatin organization, centromere dynamics, and gene regulation [28].

Furthermore, several studies indicate that TE transpositions can serve as a substrate for the emergence and mobility of satDNA, which in turn can amplify in specific chromosomal regions such as telomeric, subtelomeric, pericentromeric, or interstitial regions [14,29,30,31,32,33,34]. Although there is a great diversity of TE-derived satDNAs, they often share common features, such as monomers larger than the standard size (>500 bp) that are derived from LTRs and untranslated regions of TEs, and are located in (peri)centromeric regions [27,35].

It is known that the abundance of repeatome in the genomes of related species can diverge considerably due to heterogeneous patterns of repeat accumulation/deletion [36]. In this sense, identifying how the repeatome composition varies among species is desirable to unravel the underlying mechanisms of genome evolution in plants. Studies focused on specific genera can reveal complex patterns of diversification and adaptation that are less evident in phylogenetic analyses based on traditional molecular markers [36,37,38,39,40].

One promising plant group for this type of investigation is *Epidendrum* L. (Epidendroideae, Orchidaceae), a mega-genus of orchids widely distributed across the Neotropical region [41], which exhibits recurrent cases of hybridization [42,43]. Over the past two decades, the number of formally described species of *Epidendrum* has increased from 1000 to 1800 [44]. This mega-genus also exhibits numerous cases of adaptive radiation events [45], contributing to its diversification into plants with distinct morphological and cytogenetic characteristics. *Epidendrum* orchids show wide karyotypic diversity [46], with chromosome numbers ranging from 2*n* = 24 [47] to 2*n* = 240 [48] and GS ranging from 1C = 1.21 pg [49] to 1C = 10.1 pg [50].

In addition, the increasing availability of sequencing data for *Epidendrum* in public databases makes this genus an excellent model for repeatome evolution studies. Currently, sequencing data are available for 34 species of the genus (Table 1). Among all of them, GS data are only known for *Epidendrum rigidum* (1C = 1.21 pg; [49]), *Epidendrum nocturnum* (1C = 3.02 pg; [51]), and *Epidendrum ciliare* (1C = 3.14 pg; [52]). Despite the low representativeness of GS records for the genus, it is possible to estimate the diversity of repetitive DNA sequences in their genomes through in silico analyses. These analyses can be conducted with bioinformatics tools such as RepeatExplorer2 (RE2), which employs a graph-based clustering algorithm to identify and quantify repetitive elements from next-generation sequencing reads, even in non-model organisms without reference genomes [53].

Thus, building on the cytogenomic variation reported for *Epidendrum*, we characterized and compared the repeatome composition of the 34 species for which genomic sequencing data are currently available. Specifically, we addressed the following questions: (1) Which satDNAs and TEs constitute the *Epidendrum* repeatome? (2) How do satDNA and TE abundances vary among species? (3) To what extent are satDNA families species-specific versus shared across the genus? By providing the first multi-species repeatome comparison in *Epidendrum*, this study establishes a comparative framework to interpret repeatome turnover across closely related species, generates a curated satDNA resource for downstream analyses, and lays the groundwork for explicitly testing how repeat dynamics contribute to genome size variation and genomic evolution in this Neotropical orchid mega-genus.

## 2. Materials and Methods

### 2.1. Sequencing Data Collection for Repeatome Analysis

To characterize the repeatome in *Epidendrum*, we used sequencing data obtained from the public database of the National Center for Biotechnology Information (NCBI), representing all datasets currently available for this genus (Table 1). All genome sequences were obtained through target enrichment with the Angiosperm 353 probe set [54]. However, the off-target reads, which do not hybridize with the target genes, are still available and can be recycled to identify repetitive DNA in plants, often yielding a reduced-complexity dataset in which highly abundant repeats may be comparatively underrepresented, thereby facilitating the detection of less abundant repetitive sequences [55]. Additionally, three species outside *Epidendrum*, included as outgroups in the phylogenetic analysis, also had their repeatomes characterized using sequencing data from NCBI: *Laelia rubescens* Lindl. (SRX22571372), which was used for rooting, and *Barkeria palmeri* Schltr. (ERX7193186) and *Caularthron bicornutum* Raf. (SRX7133950), which are closely related to *Epidendrum* in the subtribe Laeliinae [56].

### 2.2. Phylogenetic Analysis

To investigate the phylogenetic relationships among the *Epidendrum* species studied herein, three molecular markers were used: The nuclear internal transcribed spacer (ITS), and two chloroplast genes, maturase K (*mat*K) and ribulose-1,5-bisphosphate carboxylase/oxygenase (*rbc*L). To further increase the representativeness and support of the phylogenetic tree generated in this study, in addition to the 34 species analyzed for repeatome composition, we added marker data from 27 additional *Epidendrum* species also available in the NCBI database (Table S1).

For species lacking available marker sequences but with genome data deposited in NCBI, we retrieved consensus sequences for the target markers through reference-guided mapping using the “Map to Reference” function in Geneious v. 7.1.3. (https://www.geneious.com (accessed on 26 August 2025)). The reference marker sequences used for this were MN332382.1 (ITS), MT518444.1 (*mat*K), and MT519153.1 (*rbc*L), all available on NCBI. When a mapped sequence showed low quality (>50% ambiguous sites) and was not available in NCBI, it was considered as missing data in the final matrix. Few differences were observed among the phylogenetic analyses for each independent marker (Figure S1), and therefore we proceeded with the concatenated analysis.

The phylogenetic analyses were conducted using Maximum Likelihood (ML) and Bayesian Inference (BI) methods. The marker concatenated matrix was prepared using Mesquite v. 3.81. Sequence alignment was carried out using the online MAFFT server [57,58], followed by a trimming step with TrimAl v1.3 [59] using the “Gappy out” method to remove poorly informative regions and manual curation in Geneious to ensure sequence length equivalence.

For the phylogenetic analyses, the best-fitting model for each molecular marker was selected based on the Akaike Information Criterion (AIC). Model selection was performed using ModelFinder [60] for ML analyses, and the *modelTest* function in the *phangorn* package [61] in R v. 4.5.2 [62] for BI. The selected models were TIM3+F+I+G4 for ITS, K3Pu+F+G4 for *mat*K, and HKY+F+I for *rbc*L in the ML analysis, while GTR+I+G was selected for all markers in the BI. ML inference was conducted using the IQ-TREE web server [63] with 1000 ultrafast bootstrap [64] replicates and 1000 iterations to assess branch support. BI analyses were performed in MrBayes v.3.2.7 [65], using four Markov Chain Monte Carlo (MCMC) chains for 10 million generations, sampling parameters every 1000 generations. A 25% burn-in was applied, discarding the initial generations to ensure the results reflected converged chains. Saved trees were summarized in a majority-rule consensus tree, and branch support was assessed by posterior probabilities (PP), with values ≥ 0.95 considered strongly supported [66,67], following Baranow et al. (2022) [68] with minor modifications.

The ML and BI consensus phylogenetic trees were visualized on FigTree v. 1.4.4. Comparison between species’ taxonomic relationships based on each tree’s results was performed using the cophylo function from the phytools package [69] in R v. 4.5.2 [62].

### 2.3. Preprocessing of Sequencing Reads

All FASTQ paired-end sequencing data were evaluated and filtered for quality using FastQC [70], integrated into RepeatExplorer2 (RE2) [53]. Reads that did not meet the criteria of ≥95% of bases with a minimum Phred quality score ≥ 10 and those containing adapter sequences were discarded. Additionally, all reads shorter than 100 bp were removed using the “Trim reads” tool, ensuring that the reads in the resulting dataset met the minimum length required for analysis. The sequences were converted to FASTA format, and the forward and reverse reads were merged into a single interlaced file, discarding incomplete pairs. The total number of reads analyzed per species in the individual analyses is provided in Table S2.

### 2.4. Individual Characterization of Epidendrum Species Repeatome Using RepeatExplorer2 and Construction of a Satellite DNA Database

Filtered sequencing data from the 34 species and the phylogenetic outgroup were analyzed independently using the RE2 pipeline provided by the ELIXIR-CZ project part of the international ELIXIR infrastructure (https://repeatexplorer-elixir.cerit-sc.cz/, accessed on 5 September 2024). For this, reads showing at least 95% similarity across a minimum of 55% of their length were grouped into clusters. Clusters with an abundance greater than 0.01% were automatically annotated and manually verified [53]. Clusters annotated as plastid or mitochondrial sequences were considered contamination and excluded from the final annotation.

For the annotation of satDNA sequences, we employed the Tandem Repeat Analyzer (TAREAN) tool [71]. This tool performs automatic annotation of satellite repeats based on the topology of the cluster graphs generated by SeqGraph [72]. All contigs with tandem repeats automatically identified by TAREAN, as well as other satellite sequences not detected by the tool but that showed typical satellite graph layouts (i.e., dense, circular graphs indicating tandemly repeated clusters), were validated according to the following criteria: (1) a satDNA consensus sequence > 100 nucleotides, which has a greater potential to form heterochromatic blocks on chromosomes; and (2) dotplot analysis using EMBOSS Dotmatcher [73] (https://www.ebi.ac.uk/jdispatcher/seqstats/emboss_dotmatcher (accessed on 26 August 2025)), showing dense line overlaps indicating a repetitive pattern in the consensus sequence.

For satDNAs automatically identified by TAREAN, the tool itself provided a consensus monomer sequence. For manually identified clusters, contigs were aligned and consensus sequences were obtained using Geneious, following Ibiapino et al. (2022) [74]. The same filtering criteria applied to TAREAN-identified sequences were used for manually annotated sequences. The monomers of the satDNAs were named independently for each species using the following pattern: a prefix of the species name (as shown in Table 1) + “Sat” + cluster number + number of nucleotides in the sequence. When pairwise sequence identity exceeded 50% between two satDNA sequences, they were classified as belonging to the same superfamily (SPF: 50–80%), the same subfamily (SBF: 80–94.9%), or as variants of the same family (F: ≥95%), as described in Ruiz-Ruano et al. (2016) [75].

Finally, candidate TE clusters in *Epidendrum* were classified based on the REXdb database of conserved protein domains [20] and annotated according to the final automatic RE2 output. All these processes were carried out independently for each species to characterize the species-specific repeatome profiles of the orchids.

### 2.5. Comparative Analysis of the Repeatome Composition in Epidendrum

To answer the second and third questions of our study regarding differential abundance and sharing of repetitive elements among species, we performed a comparative analysis of the repeatome composition of the 34 *Epidendrum* species and the phylogenetic outgroup taxa. For this, forward and reverse read sequences from each taxon were interlaced in RE2 and randomly sampled in sets of 500,000 reads per species, following the normalization recommendations of Novak et al. (2020) [53]. Interlace reads from each species were identified with a different prefix, all datasets were then concatenated and finally clustered using the comparative analysis option with default settings (Table 1).

The comparative analysis used the same parameters applied in the individual analyses. The main distinction in this step was the inclusion of a custom satDNA database based on the individual *in silico* annotation of the 34 *Epidendrum* species described in the previous topic (Table S3). In cases where RE2 annotated more than one satDNA sequence from the custom database to a single supercluster, we adopted the following criteria to determine the final annotation: (1) if one satDNA showed a similarity score at least twice as high as any other, it was retained as the final annotation, as per RE2’s standard procedure; or (2) if similarity scores among the satDNAs were very similar, we aligned the consensus sequences and grouped them into the same superfamily when they shared at least 50% identity, following Ruiz-Ruano et al. (2016) [75].

If the same class of satDNA was present in different species, the corresponding reads from those species were grouped into the same cluster due to sequence similarity. Conversely, clusters containing reads from only one species were considered taxon-specific repeats [53]. Since quantitative information derived from read counts in clusters is also available, these data were used to analyze differences in the abundance of repeats among the species analyzed.

## 3. Results

### 3.1. Phylogeny of Epidendrum

The final matrix consisted of 2392 characters divided into three partitions: 1–653 (ITS), 654–1458 (*mat*K), and 1459–2392 (*rbc*L), representing 445 distinct patterns, of which 184 sites were informative. We observed few differences between the results of the ML and BI trees, mainly related to poorly supported dichotomies in the ML tree, which were displayed as polytomies in the BI tree (Figure S2). The only species with notably divergent placement between the two trees was *Epidenrum campestre*, which was associated with different groups of species (Figure S2). Still, the ML phylogenetic tree generally showed higher branch support than the BI tree (as seen in the clade of *E. igneum*, *Epidendrum coclidium*, and *Epidendrum xanthinum*; Figure S1). For this reason, we chose to show the comparative organization of repetitive elements based on the ML phylogeny results in the following sections.

### 3.2. Individual and Comparative Analysis of Repeatome Composition in Epidendrum

In our individual analyses conducted using RE2 with data from the 34 *Epidendrum* species, we observed that repeatome profiles varied significantly among species. The proportion of satDNA ranged from 1% in *Epidendrum sophronitoides*, *Epidendrum longicaule*, and *Epidendrum parkinsonianum* to 99% of the repetitive fraction in *Epidendrum rivulare* and *Epidendrum difforme*. In contrast, TEs ranged from 0.3% in *E. rivulare* and *E. difforme* to 96% of the repeatome in *E. longicaule*, according to manual curation and RE2 automatic annotation (Table S2). These results refer to the repeatome characterization performed independently for each species. Moreover, by summing all satDNAs identified across the 34 species in the individual analysis, we obtained a dataset of 208 satDNAs, which were included in a custom database for *Epidendrum*. This database was then used to support cluster annotation in the comparative analysis.

**Figure 1:**
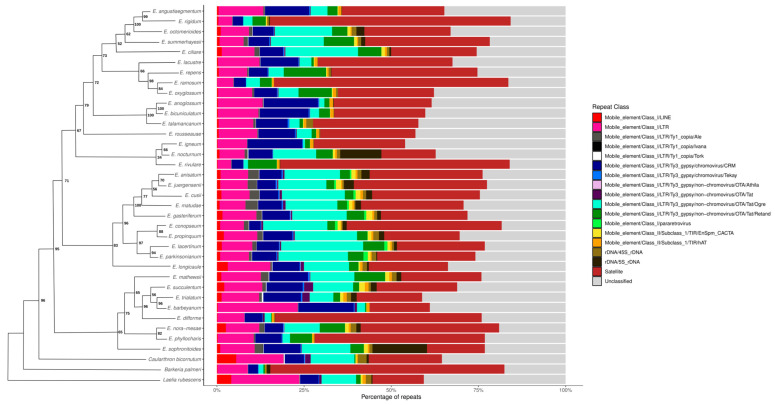
Comparative distribution of repetitive elements across *Epidendrum* species. Bars represent the percentage of reads of different classes of elements, identified according to the color scheme in the legend (right). The schematic phylogenetic tree (left) is derived from the ML topology (simplified from Figure S2 for clarity), with node labels indicating bootstrap support values based on likelihood analysis.

The comparative analysis of the repeatome from the 34 *Epidendrum* species identified 160,041 clusters from 2,495,238 analyzed reads, of which 775,261 (31%) corresponded to repetitive element sequences (Figure S3). The clustered sequences were automatically annotated by RE2, which detected 50,001 organellar reads that were excluded from subsequent analyses, along with other unannotated sequences from small clusters. Summing the automatic annotations from RE2, TAREAN, and manual satDNA annotations, we identified 23,121 ribosomal DNA reads (3% of the repeatome), 374,429 reads associated with satDNA (48% of the repeatome), and 377,711 reads associated with TEs (49% of the repeatome) in *Epidendrum*. Among these sequences, the number of reads classified as satDNA varied by up to ninefold between *Epidendrum phyllocharis* (34,569 reads) and *Epidendrum oxyglossum* (3708 reads), while reads classified as TEs varied by up to elevenfold between *E. phyllocharis* (19,794 reads) and *E. rigidum* (1731 reads) (Figure 1).

### 3.3. Characterization of Satellite DNA Composition in Epidendrum

With the aid of the custom satDNA database previously built for the genus in the individual analysis, a total of 73 clusters (abbreviated as CL) were annotated as satDNA in *Epidendrum* in the comparative analysis, with the number of shared monomers per species within this total ranging from 37 in *E. longicaule* to 58 in *Epidendrum ramosum* (Table 2). Of all these clusters, 48 represent unique monomer sequences, while the remaining 28 are distributed across 18 superfamilies since consensus sequences shared at least 50% identity (Table S4). Among these superfamilies, SPF2 is the most abundant, representing 19% of all satellite sequences in *Epidendrum* and including the monomers EoctSat57-510 (from *Epidendrum octomerioides*) and EdifSat97-156 (from *E. difforme*). The only species-specific satDNAs identified in this analysis were CL430, present exclusively in *Epidendrum gasteriferum*, and CL359 and CL382, found only in *E. rigidum*.

**Figure 2:**
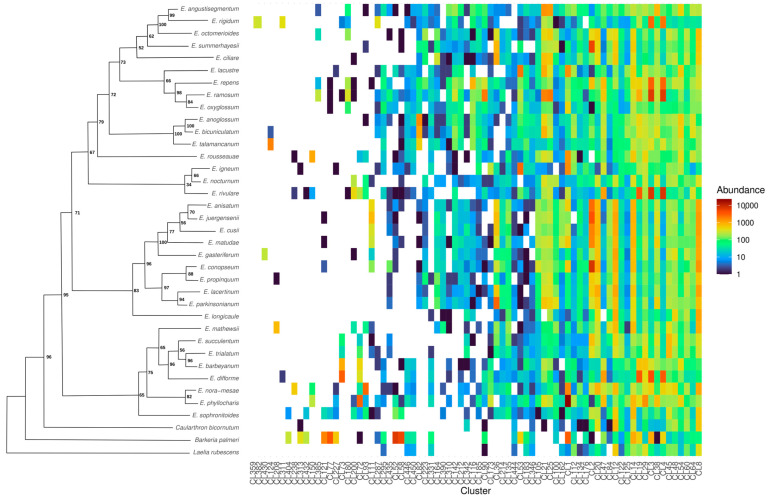
Sharing and abundance of the satellite DNA set identified *in silico* in *Epidendrum*. The graph shows the abundance of satDNA cluster reads per species, with values transformed using a base 10 logarithms (log10). This transformation was applied to reduce disparities between the highest and lowest values, making relative differences among species easier to visualize. The legend’s color scale reflects cluster abundance, where warmer colors indicate higher values, cooler colors indicate lower values, and white gaps indicate cluster absence. The schematic phylogenetic tree (left) is derived from the ML topology (simplified from Figure S2 for clarity), with node labels indicating bootstrap support values based on the likelihood analysis.

**Figure 3:**
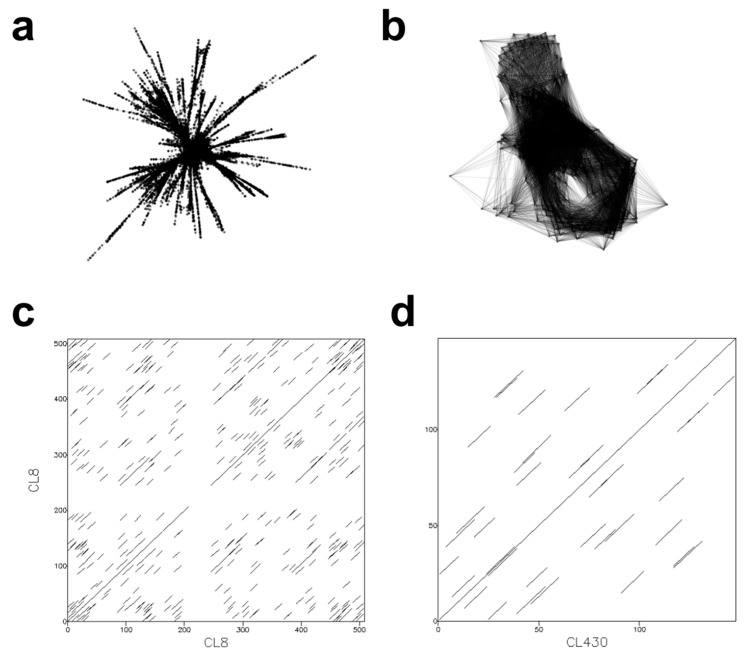
Graph layouts for two of the most and least shared satellite DNA clusters in *Epidendrum*. (**a**) Shows SeqGraph layout for CL8 from SPF2, the most abundant and widely shared cluster; (**b**) Shows SeqGraph layout for CL430, which is specific to *E. gasteriferum*. (**c**) Dot plot graphs generated for the consensus sequence of CL8. (**d**) Dot plot graphs generated from the consensus sequence of CL430.

The other satDNA monomers and non-specific superfamilies were broadly distributed across the 34 *Epidendrum* species, allowing us to evaluate how these elements are shared across them (Figure 2). We observed that most clusters exhibited characteristic satDNA graph layouts, with high read density connections between cluster vertices. Moreover, dot plot graphs for these satDNAs showed dense overlapping lines, indicating a repetitive pattern in the consensus sequence of the monomers. Structural analysis of the identified satDNA clusters revealed that despite the substantial variation in repeatome abundance (15.5% to 59%; Table 2), the monomer sequences are highly conserved across species. The identified monomers range from 75 bp to 1114 bp, with a predominant length of 170 bp and an overall mean of 257 bp (Table S3). For instance, the most abundant superfamily SPF2, exemplified by the widely shared cluster CL8, exhibits high structural stability across the genus, contrasting with the few species-specific satDNAs identified (e.g., CL430 in *E. gasteriferum*; Figure 3).

### 3.4. Characterization of Transposable Element Composition in Epidendrum

**Figure 4:**
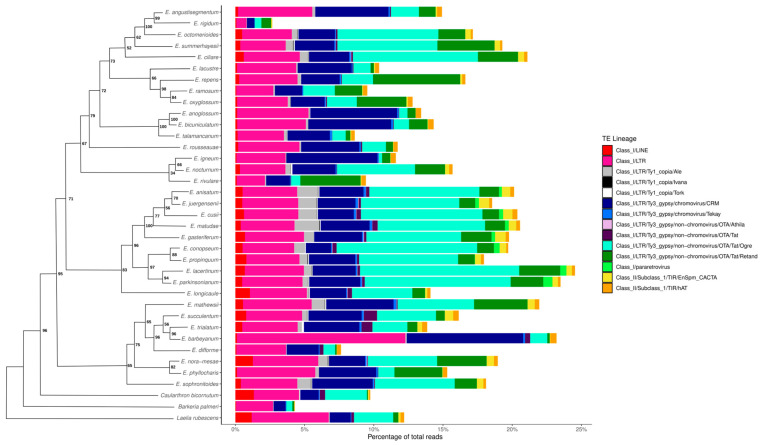
Diversity of transposable elements in *Epidendrum*. Comparative distribution of TE read abundance across *Epidendrum* species. The different lineages are identified according to the color scheme in the legend. The schematic phylogenetic tree (left) is derived from the ML topology (simplified from Figure S2 for clarity), with node labels indicating bootstrap support values based on likelihood analysis.

Considering the distribution of TEs within the *Epidendrum* repeatome (Figure 4), the comparative analysis indicated that 97% of the identified TEs belong to Class I retrotransposons, while the remaining 3% correspond to Class II elements. Among Class I elements, LTR retrotransposons were the most abundant order, accounting for 97% of all retrotransposons, with the remaining 3% represented by long interspersed elements (LINEs). Regarding Class II TEs, 51% of the elements were classified as terminal inverted repeats (TIRs) of the EnSpm_CACTA type, and 49% belonged to the hAT family.

**Figure 5:**
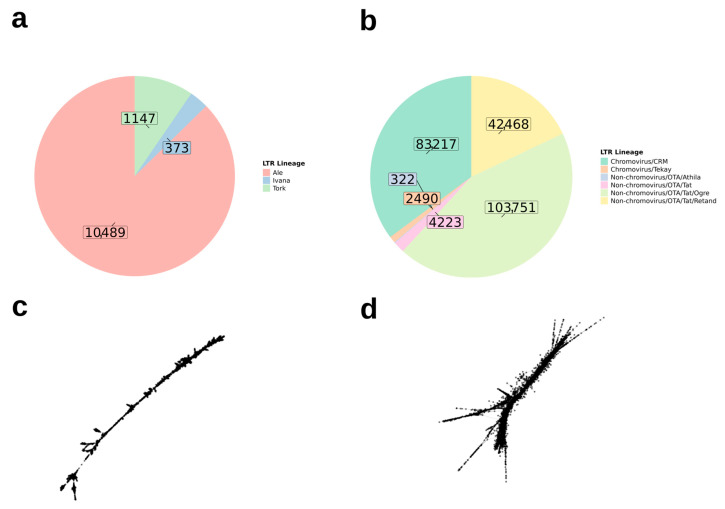
Most abundant lineages of long terminal repeat retrotransposons in *Epidendrum*. (**a**) Pie chart showing the total number of reads of Ty1-Copia lineages (Ale, Ivana, Tork) identified in *Epidendrum*. (**b**) Pie chart showing the total number of reads of Ty3-Gypsy Chromoviruses (CRM, Tekay) and non-Chromoviruses (Athila, Tat, Ogre, Retand) lineages identified in *Epidendrum*. (**c**) SeqGraph layout of a typical Ty1-Copia cluster (CL109). (**d**) SeqGraph layout of a typical Ty3-Gypsy cluster (CL9).

Moreover, we observed that among the two main LTR retrotransposon superfamilies, Ty3-Gypsy was approximately 20× more abundant than Ty1-Copia (236,471 and 12,009 reads, respectively; Figure 5), whereas only the Ale, Ivana, and Tork lineages were detected for Ty1-Copia. For Ty3-Gypsy, the identified lineages included Chromoviruses CRM and Tekay, as well as Non-Chromoviruses such as OTA/Athila, Tat, Ogre, and Retand (Figure 5a,b). At the individual level, Ogre was the most abundant LTR lineage across all species, while Ivana was the least frequent (Figure 4). Representative graph layouts of selected Ty1-Copia and Ty3-Gypsy clusters, CL109 and CL9, respectively, illustrate the typical structural organization of these elements in *Epidendrum*, characterized by dense, linear-like topologies (Figure 5c,d).

## 4. Discussion

Our analysis of repetitive DNA elements in the mega-genus *Epidendrum* provided an opportunity to uncover potential genomic patterns across species with contrasting biogeographic histories and a wide geographic distribution, occurring from the United States to southern Brazil [76]. We found that although some satDNAs are heterogeneously distributed among the species, most repeat families are shared across species, supporting the library model proposed by Salser et al. (1976) [12]. While the repeatome composition cannot yet be directly linked to differences in total nuclear DNA content without GS measurements, our analysis offers a preliminary insight into the genomic landscape of the genus. Moreover, although complete genome sequencing has not been performed for any species, it was possible to estimate the amount of repetitive elements present in their repeatomes using enriched sequencing data publicly available by leveraging off-target reads characterization. Although each technique has distinct biases, both can recover the same repeat families, including low-abundance repeats [55,77]. Nevertheless, it is important to note that biases in satDNA and TE frequencies may occur due to genomic library enrichment, which could partly explain the unusually high satDNA fractions observed in *Epidendrum rivulare* and *Epidendrum difforme*. In this sense, both target-capture sequencing (as used here) and genome skimming are viable approaches for repeatome characterization, but enrichment can affect the estimated absolute abundances of repetitive sequences.

With regard to phylogeny, the species relationships obtained here are mostly in agreement with those recovered by Granados Mendoza et al. (2020) [56], who indicated that the genus *Epidendrum* is divided into two main clades, called Clade A and Clade B, shortly after the divergence of *Caularthron bicornutum*. Clade A includes one group with *E. sophronitoides* as the sister species to *E. nocturnum*, and another group that includes *Epidendrum mathewsii*, *Epidendrum succulentum*, and *Epidendrum trialatum,* while Clade B comprises the another 13 sampled species. Although their overall phylogenetic structure agrees with previous classifications, the recovery of *E. nocturnum* within Clade A directly contrasts with earlier studies (such as [78]), where this species was traditionally placed in Clade B. In the present study, the same species of Clade A examined by Granados Mendoza et al. (2020) [56] are also grouped within a single clade together with additional species investigated in our study (*Epidendrum barbeyanum*, *E. difforme*, *E. phyllocharis*). However, *E. nocturnum* was grouped in the second clade following previous classifications [78], together with all other species analyzed here. This discrepancy in the position of *E. nocturnum* between studies may be primarily related to intraspecific variation [79,80,81]. Additionally, the inclusion of different species may also have influenced the repositioning of *E. nocturnum*, since sampling composition directly affects the stability and resolution of clades.

### 4.1. What Are the Main satDNA and TE Components, and What Are Their Abundances Across Epidendrum Species?

When comparing the distribution of satDNA clusters among species, we found that, although there is considerable variation in the abundance of these elements among them, the monomer sequences tend to be shared among different lineages (Figure 3). Sequences of satDNA are known to evolve rapidly, exhibiting high mutation rates and variation in abundance and chromosomal location. Nevertheless, the mechanisms underlying these changes are still debated, with unequal crossing-over, gene conversion, and rolling-circle replication among those proposed [10,35,82].

Considering TE lineages, Class I elements are more prevalent in *Epidendrum* repeatomes than Class II elements, as is the case in most plant genomes [18]. Although Class I TEs are largely intergenic, most Class II TEs are preferentially found within or near genes. Thus, Class I elements are generally known to contribute more significantly to GS variation in plants, while Class II elements are often involved in generating allelic diversity [83]. Although a direct correlation analysis was not possible due to the limited GS data available for the genus, we hypothesize that the TE diversity observed herein could be a potential source of GS variation among *Epidendrum* species. This hypothesis provides a framework for future studies combining broad GS estimations with genomic characterization.

Among the Class I elements in *Epidendrum*, most are classified as LTR retrotransposons, which was expected given that these are the most abundant group of TEs in plants [18]. Furthermore, the LTR lineages found in *Epidendrum* reflect the main evolutionary lineages of the Ty3-Gypsy and Ty1-Copia superfamilies [25]. A notable observation from our data is that Ty1-Copia is nearly 20 times less abundant than Ty3-Gypsy (Figure 5). It is known that among the two superfamilies that compose autonomous LTR retrotransposons in plants, Ty3-Gypsy is usually more abundant in genomes than Ty1-Copia [84,85,86], which is consistent with our data. Although they share similar structural characteristics, Ty3-Gypsy and Ty1-Copia differ both in their gene sequence composition and in the organization of the domains within the *POL* gene that they encode [87].

In *Helianthus*, it was found that the proliferation of Ty1-Copia elements in the genomes is much lower than that of Ty3-Gypsy [88]. Moreover, the scale of increase in copy number of these elements differs considerably among the hybrid species compared to the average value of the parental species. This may happen due to transcriptional silencing via DNA methylation and chromatin modification, and the disruption, potentially caused by hybridization or environmental stress, could induce a form of genome shock. On the other hand, in some cases, Ty1-Copia can be the most abundant LTR component, accounting for up to 40% of the TEs in the species *Linum usitatissimum* L. [84] and 10.7% in *Cucumis sativus* L. [89], for example. In this context, differences in the degree of LTR retrotransposons proliferation among *Epidendrum* species may reflect random dynamics of Ty1-Copia and Ty3-Gypsy element activation. They may also be influenced by environmental conditions of the habitats where the species occur, as well as by hybridization events, which are recurrent in the genus [42,43,90]. This may occur because genomic shocks [91] caused by environmental stress and hybridization can loosen gene expression regulation, inducing TE activation and leading to rapid genetic and epigenetic changes, including chromosomal rearrangements. As a consequence, this set of changes may contribute to the stabilization and diversification of new species [88,92,93,94,95].

Considering the Class II elements in *Epidendrum*, we observed that they include only two TIR superfamilies: EnSpm_CACTA and hAT. It is known that only five of the seventeen Class II TE superfamilies characterized to date have been found in plant genomes: EnSpm_CACTA, Mutator, PIF/Harbinger, hAT, and Tc1/mariner [83]. Therefore, the identification of EnSpm_CACTA and hAT in the *Epidendrum* repeatomes is consistent with what has been reported in the literature for Class II TEs in plants, while the absence of the other superfamilies may reflect either their elimination in these species or a sampling bias, considering that our data do not include all representatives of *Epidendrum*, but only those for which sequencing data are currently available.

### 4.2. To What Extent Are satDNA Families Species-Specific Versus Shared Across the Genus?

Although our dataset represents less than ~2% of the total species diversity within *Epidendrum*, the repeatome characterization of the 34 species described here has already shown that satDNAs and TEs can vary considerably among them, potentially impacting repeatome differentiation and, consequently, genomic differentiation.

According to the library hypothesis of satDNA evolution, related species may share a set of conserved satDNA sequences over long evolutionary periods, which are mostly subject to quantitative changes [10,33,35,96]. In several studies examining the composition and variation in satDNA sets in different related species within the same genus, this pattern is recurrent, and significant expansions and contractions in satDNA content are observed among closely related lineages over short evolutionary timescales, as reported in both animal and plant groups [34,35,38,97,98,99]. For example, in *Asclepias* (Apocynaceae) the abundance of satDNA ranges from 0.98% to 7.73% among species, with some satDNA families being correlated with phylogeny and geographic distribution, indicating that variation accompanies evolutionary diversification [100]. Similarly, our results suggest that the set of 73 shared satDNA clusters identified in *Epidendrum* is part of an ancestral “satellite library” that has been present in all 34 sampled species. Exceptions to this pattern are CL430, found only in *E. gasteriferum*, and CL359 and CL382, exclusive to *E. rigidum*. Throughout evolution, independent expansion and contraction of these satDNAs in different lineages may have contributed to the diversification of the genus.

Additionally, there does not appear to be a strict correspondence between phylogenetic proximity and repeatome profile similarity. For example, closely related species in the tree, such as *Epidendrum igneum* and *E. nocturnum*, exhibit notable differences in the proportion of satDNAs and TEs, which may indicate independent events of gain and loss of repetitive elements following the potential divergence from *E. rivulare*. This phenomenon suggests that repeatome evolution in *Epidendrum* may be influenced by factors specific to the natural history of each species, such as adaptation to different habitats, internal mechanisms of genome regulation, and even physiological traits which may converge toward similar repeatome patterns even in taxa that are more distantly related in the phylogeny [101].

## 5. Conclusions

Our study provides the first multi-species characterization of repetitive DNA in the Neotropical mega-genus *Epidendrum* and establishes a comparative foundation for future investigation into how repeatome dynamics may contribute to genomic diversification. Overall, our results indicate that *Epidendrum* repeatomes are highly heterogeneous, even among closely related species, which often display contrasting satDNA and TE profiles. Despite this heterogeneity, repeatomes are consistently dominated by Class I LTR retrotransposons, particularly Ty3-Gypsy lineages, and by a set of widely shared satDNA families. The prevalence of shared satDNAs across species is consistent with the satDNA library hypothesis, in which conserved sequence variants persist over evolutionary time while their genomic abundance changes markedly among lineages. Accordingly, the uneven distribution of satDNA and TE clusters among species supports a scenario of lineage-specific amplification and loss, pointing to rapid repeatome turnover in *Epidendrum*. Although genome size data are currently available for only a few species, the resources generated here, including a curated satDNA database and repeat annotations across 34 taxa, provide an explicit roadmap for future work integrating broader genome size sampling, cytogenetic mapping, and ecological or demographic data to test the drivers of repeat expansion and contraction.

## Figures and Tables

**Table 1:** Sequencing data of all *Epidendrum* species currently available in NCBI.

NCBI ID	Species	Prefix
ERX7192163	*Epidendrum angustisegmentum* (L.O.Williams) Hágsater	Eang
SRX7133951	*Epidendrum anisatum* La Llave & Lex.	Eani
SRX22571358	*Epidendrum anoglossum* Schltr.	Eano
SRX22571359	*Epidendrum barbeyanum* Kraenzl.	Ebar
SRX22571360	*Epidendrum bicuniculatum* Hágsater & E.Santiago	Ebic
SRX7133952	*Epidendrum ciliare* L.	Ecil
SRX7133937	*Epidendrum conopseum* R. Br	Econ
SRX7133953	*Epidendrum cusii* Hágsater	Ecus
ERX7193246	*Epidendrum difforme* Jacq.	Edif
SRX7133954	*Epidendrum gasteriferum* Scheeren	Egas
SRX22571361	*Epidendrum igneum* Hágsater	Eign
SRX7133955	*Epidendrum juergensenii* Rchb.f.	Ejue
SRX7133935	*Epidendrum lacertinum* Lindl.	Elac
SRX22571362	*Epidendrum lacustre* Lindley	Elau
SRX7133936	*Epidendrum longicaule* (L.O. Williams) L.O. Williams	Elon
SRX7133938	*Epidendrum matthewsii* Rchb.f	Emah
SRX7133939	*Epidendrum matudae* L.O.Williams	Emau
ERX7193247	*Epidendrum nocturnum* Jacq.	Enoc
ERX7193201	*Epidendrum nora-mesae* Hágsater & O.Pérez	Enor
SRX7133941	*Epidendrum octomerioides* Schltr.	Eoct
SRX22544933	*Epidendrum oxyglossum* Schltr.	Eoxy
SRX7133942	*Epidendrum parkinsonianum* Hooker	Epar
SRX22571363	*Epidendrum phyllocharis* Rchb.f.	Ephy
SRX7133943	*Epidendrum propinquum* A. Rich. & Galeotti	Epro
ERX7193248	*Epidendrum ramosum* Jacq.	Eram
ERX7193250	*Epidendrum repens* Cogn.	Erep
ERX7193245	*Epidendrum rigidum* Jacq.	Erig
ERX7193249	*Epidendrum rivulare* Lindl.	Eriv
SRX22571365	*Epidendrum rousseauae* Schltr.	Erou
SRX7133944	*Epidendrum sophronitoides* F. Lehm. & Kraenzl.	Esop
SRX7133946	*Epidendrum succulentum* Hágsater	Esuc
SRX7133947	*Epidendrum summerhayesii* Hágsater	Esum
ERX7193031	*Epidendrum talamancanum* (J.T.Atwood) Mora-Ret. & García Castro	Etal
SRX7133948	*Epidendrum trialatum* Hágsater	Etri

NCBI ID corresponds to the accession code of the sequencing dataset for each species.

**Table 2:** Comparative summary of satDNA identified in *Epidendrum* species *in silico*. The number of shared satDNA clusters refers to sequences classified by RE2 (with automatic and manual annotations using the *Epidendrum* satDNA custom database) that are shared with other species of the genus (Figure 2). The third column indicates the total satDNA abundance (%) in each species dataset, and the last column shows the total number of reads analyzed in RE2.

Species	No. of Shared satDNA Clusters	Total satDNA Abundance (%)	Total Reads Analyzed in RE2
*E. angustisegmentum*	55	29.39	64,728
*E. anisatum*	50	32.38	65,012
*E. anoglossum*	54	28.06	64,350
*E. barbeyanum*	47	17.28	64,175
*E. bicuniculatum*	52	26.10	64,664
*E. ciliare*	48	24.54	64,766
*E. conopseum*	50	44.44	64,592
*E. cusii*	46	30.90	64,106
*E. difforme*	47	59.22	64,346
*E. gasteriferum*	47	24.82	64,446
*E. igneum*	47	26.15	64,690
*E. juergensenii*	45	38.17	65,008
*E. lacertinum*	47	25.20	64,416
*E. lacustre*	49	38.64	64,990
*E. longicaule*	37	22.69	64,640
*E. mathewsii*	44	22.98	64,566
*E. matudae*	50	29.41	65,036
*E. nocturnum*	48	15.56	65,484
*E. nora-mesae*	51	39.68	64,272
*E. octomerioides*	51	24.70	64,870
*E. oxyglossum*	53	27.53	35,926
*E. parkinsonianum*	47	28.17	65,092
*E. phyllocharis*	52	48.76	129,334
*E. propinquum*	47	21.64	64,664
*E. ramosum*	58	67.18	64,820
*E. repens*	56	41.89	64,834
*E. rigidum*	50	69.03	65,004
*E. rivulare*	54	65.81	65,026
*E. rousseauae*	49	27.29	65,378
*E. sophronitoides*	51	16.57	64,814
*E. succulentum*	42	23.07	64,600
*E. summerhayesii*	46	35.72	64,924
*E. talamancanum*	49	30.20	64,266
*E. trialatum*	43	18.75	64,452

## Data Availability

The original contributions presented in this study are included in the article/Supplementary Materials. Further inquiries can be directed to the corresponding authors.

## Supplementary Materials

The following supporting information can be downloaded at https://www.mdpi.com/article/10.3390/genes17020161/s1. Figure S1: *Epidendrum* phylogenetic trees inferred from individual molecular markers; Figure S2: *Epidendrum* phylogenies based on Maximum likelihood (ML) and Bayesian Inference (BI) analysis; Figure S3: Krona chart of *Epidendrum* repeatome; Table S1: GenBank accession numbers for molecular markers (ITS, *mat*K, and *rbc*L) sequences used in the *Epidendrum* phylogenetic analyses; Table S2. Individual repeatome analysis. The total read counts and relative abundances of major repeat classes in the individual repeatome analyses of each *Epidendrum* species (satDNA, TEs, rDNA, and other repeats as rDNA 5S and 45S); Table S3: *Epidendrum* satDNA consensus sequences identified in the individual repeatome analyses (custom satDNA database); Table S4: Classification of satellite DNA clusters superfamilies in *Epidendrum*.
