# Supplementary material for: The Repeatome in the Mega-Genus Epidendrum L. (Epidendroideae, Orchidaceae): An In Silico Comparative Analysis
Source: Genes (Basel). 2026 Jan 30;17(2):161. doi: 10.3390/genes17020161 (PMC12940908; doi:10.3390/genes17020161)
Supplement: Supplementary file 1 [file genes-17-00161-s001.zip › Humberto et al_Table S3.pdf]

The Repeatome in the Mega-Genus *Epidendrum* L. (Epidendroideae, Orchidaceae): an *In Silico* Comparative Analysis

Table S3. *Epidendrum* satDNA consensus sequences identified in the individual repeatome analyses (custom satDNA database)

Satellite DNA	Nucleotide Sequence	Length (bp)
EangSat56-144	TAAATCCCCACCACCACCA<br>TCACCTTCTCCTCCACCTCC<br>CTATCACTATAAGTCTCCTC<br>CTCCCCCATCTCCCTCACCA<br>CCTCCTCCATACTACTACA<br>AATCACCTCCCCCACCATC<br>ACCATCTCCTCCTCCACCAT<br>ATCATTA	144
EangSat238-384	AGTTCCACAAGGAGGAGAG<br>AGACGGCGGTCGTGGGAAT<br>AAGGAATGGGGAGGCAGC<br>TACAAGAAGAAGAAGAAT<br>GAGGAAGAGGATGAGGGT<br>GGTTTTGGTGGCAATGGCA<br>AGAAGAAAGAGGTTGGTG<br>ATAGCGAGAAGAAAGAGA<br>GGGGAGAGTGTAGTCGTGG<br>TGTAAAGAATATAATGGAT<br>GGAGTACGCTGGCAGAAGG<br>AGGGAGCTGATGACGAGA<br>AGAAGCAGAAGAAGGAGA<br>TGGAGTACTGGAACGGTGG<br>TTTTTGGTTGGAAGAGGAG<br>GGAGGTAGTGGCCACGGGA<br>AGGAGGAGGGTGGTGGCA<br>ATCGCAAGAAAAACGAGG<br>AGGAGAAGCGAAGCAGTG<br>GTCGTGATGAGAAGAAAAA<br>AGTGAGAGGTCATG	384
EangSat16-422	GRKKKRRGSKGSGRKKKSR<br>RSGNNNNNNNNNNNNNNN<br>NNNNNNNNNNNNNNNNNN<br>NNNNNNACCTCCTNAYACA<br>CCCCTACCMCCGCATACGC<br>TMTWCGMATTTCTCACTCT<br>ACTCCCTCCYYCCCCTCCCC<br>CTCCCCCCCCCCCCTCTCTC<br>TCTCCCTCTCTCTCTCTCTC<br>TCTCCNCTCCTCTCAGAGA<br>GGGAAGGGGGGAGCTNTCT<br>CCTCCCCCCCCCCCCTCGN<br>NGAGAGGGAGAGAGAGGG<br>RGAGAGAGAGAGAGAGGG<br>AGGGAGAGGGARAGRGRG<br>GGAGRGAGRGAGRGAGAG	422

	AGAGAGAGAGAGGGAGGG<br>RGRSATAKAGAGAGAGAAA<br>GAGAGRGAGGGGGGGRGS<br>GGGRGGGGGGGGGGGGG<br>GAGGGGAGGGGGGGGGG<br>GGGGGAGTGGGAGGGGGG<br>GGCGGCGGGGTG	
EangSat10-824	CATATATGATGCCTGACSSC<br>KGRWYYTKRMKRWTYSGS<br>GGSAGMAAATTCWAAAAH<br>CHAAAATTMAAAAATAAA<br>TGATAATAAATTCCCTCTTT<br>ATTTAATTCAAAAAAAAAM<br>MAAAAMAAAAATCTYTAA<br>AAAACAAAAAAMAAAACA<br>CCCGTGGCCTAGGGAWTAT<br>TAAAGGTAGAWATKGTTA<br>AATTGGTTGKTTAATTTTKT<br>AAATTAGGAATGGGAGATT<br>GGTGGCTGGCTATTTTAAG<br>ATTTGGGAATATCCGGGAA<br>GTCCACTATCGGAGGGGCT<br>AGGGGGCTGCCCTTGCGCC<br>GGAGTTCCTTGGCCGSCGC<br>CTCTGAGGGGGTACCCGGC<br>GGCCCTATATAGGCCGCCG<br>GGTAATCCTTGGAGGTGCA<br>CTCCCSCCTCCTCGGACGGC<br>CCCTTCCCCAGACCACCCG<br>ACACCCCCCACCGGSCMAC<br>CCGAAAGCCCCCGCCCCCC<br>CTCCTCCCCCCACAAAAAC<br>CCCCCCCCCCCAATTTCCTA<br>AAATAACCCCCCCCCACCC<br>CTTCCCCTTCCCAAAACCC<br>ACCCCCTCCCCTTTTGAWT<br>TTCCCRAAAWTTCCTTCYT<br>TTTGAAWRGGCCGCCCTCC<br>YTCCTCCCCCCCCTCCHTTT<br>CCCTACCATCCCCCTTCMTT<br>CYCCCTCCCTHCCCATTTYC<br>CYCTCAGMTMAGCTCCTCS<br>ACGWTYGTTGATTTGAWAT<br>AATKGSTACCGWGGTTATT<br>NACTTTTNCYWAWTCGGA<br>RAWYYTWACCCTGTGGAT<br>TTCCCGGACATTCCCTCCAC<br>TTGGAGTGGCAGGCCACCT<br>TCCTCCCTCCCCCTCCTTCC<br>CCTTCCTACTTTCCTTATAT<br>AGCC	824

EangSat37-570	CACCGCTCCTCCAAAATAT<br>GAAGAAGAAGAAGAAVAT<br>GGTAAAAATSAAAAWTAV<br>GSHGAVAAARAWGTCACT<br>ACAGGAATCAAGTTAATTA<br>CCGACGGAATTTTTCTGAC<br>GGATTTTAGTCCGTCGGAG<br>TCGGCGACGGCGGCGGCGT<br>CGACGGCGGCGGCGGCGAC<br>GGCGGCCGGAAGGATGCCG<br>AGGGAGGGAGAGAGGAGC<br>AGAGGCGCAGAGGGGAGA<br>GGAAAKCKAGAGAGAAAA<br>NAGAATGGTTTCGGGTTGT<br>GACCTAATTCCTATTTAATT<br>ACTGACGGAAAATCCGTCG<br>GAATTTCCGTCAGTAAATT<br>CTAAATAAAAACTGGGTSA<br>AATCCCRGAACCCGCATTT<br>CTCGYTTCYCGTTCTCTCTC<br>CCTCTCCCTCTCCCCCCCCC<br>CGCGCCTCTSYCYCTCTCTC<br>CCTCCCTCGACTCATCCTTC<br>CGGCCGCCGCCGCCGCCGC<br>CGCCGCCGCCGCCGCCGCC<br>GCCGYCGCCGTCKTCCGCC<br>GCSMMGYCCAAAAAACTT<br>CCGGCTAGGTATTCCATTTT<br>TTATTCATTTACCACCTTTC<br>ATATTTATTTCAAGTGAAA	570
EaniSat362-112	GGGTTTCAATGTCTAGTTTC<br>GAATGGGAGATCGGACGCG<br>ATCTCCAGCTCATCCGACT<br>GTAACCAGGCAAGGGATCA<br>ATCTGTAGGGGACAGAAAG<br>ACCAACTCGAAGCAGT	112
EaniSat55-225	ATGAGGAGGGAGATCCATC<br>GAGGGTTTACTCTGATCCT<br>AATAGTCAGGGATCCGTAG<br>GGGAGAGGTGTCAACTCAG<br>GGCACTACTCTGATCCTAT<br>GAGAATCCTAAAAAGGGGC<br>GACTGGTTGGGGGGTATCC<br>ACTAGAGGCTCATACCGGT<br>CCAATCAGGGCTAGGACCA<br>ACAAGGAAAGTGTCCACTA<br>AGGGTCTACACTAACCTTG<br>GGTCAAAAGGTGACTA	225
EaniSat99-132	CCACAGGATGTGAATCCGC<br>ATGGTCTTCCTGTCTCCCTG<br>GTTAGGACGTAAGTCCGCA<br>TGGTTGCCCTAACCATGAG	132

	TCGTTAGGGTTGAGATGTGAATCCGCATGGTATTCTCACTCCCTTTCACTCTTTT	
EaniSat262-119	TCACTTCAGGTTGGTCTTTCTGTCCAGCTACAGATTGACCCCTTGTCTGGTTGTTCGAAGATTAGGCTGACTTCTACCGGTCACCACCTATTCCTAGATCAGGCATTGGACACCTTACT	119
EaniSat283-100	CTCTCCTATAAGATCTCCTGCTGACTATGAGGACCCGATAAGTTCTCTCCTCATTAGTCATTGTTATCTTCTTCAGGAATCTCATAGGACCCGATAAGTT	100
EaniSat7-235	CAATCTTCTCCAAAYWRKWYSSWMAMCCTWAGTGRGGCCCACTTGACTTAAATAGGTCAAAGTTAGGGTTTTTGGTCAAACAAGCTCAAAGTTTGACCAAACTNAGTCCCAAACTTTTCTAGTAGGCTTCTAGTANNTGTGAAAGTTTGTGAGAAGTTTGGTCAAATTTTGAGCTGTTTGACCAAACACCCTAACTTTGACCTATTTAGGTCAAATTGGCCCMACTTAGGGTTTG	235
EaniSat19-233	CTCCAACTTGGACCAAACTTCCCACAAACCTTCACACATACACTAGAAGCCTATTAGACAAGTTTGGGACCCATTGGGTCTGGTCACCCTTTGAGCCTAGTYAAGAGTAAAACCCTAAGTGGGGCCCAATTGACCTAAATAGGTCAAAGTTAGGGTGTTTAGTCAATGGGTYCCAAACTTGTCTAATAGGCTTCTAGTGTATGTGTGAAGGTTTGTGGGAAGTTTGGTCCAAT	233
EaniSat68-520	CAAATWWWTWWARTAAATAATTTATTTTATYTTTWTTAWTWATTATWTNANANTTGAGAGATTGAMTWAGTGAGAGAKNNNGAANNAGATGGAKTGABAGATAWTTACGAWTAGNAGATTGATAAAATTTTTCTGKCATAATTTTAACTTCAATATTCCCTGCGTCGTTGTCGCTAWTGATTTMTC	520

	CCACTCCNAATCTCTCTCTC<br>TCCCTCCTTCCTCTCCTTCC<br>CCTCTCTCTCAACCCCTCCT<br>CCVTTCTCTATCTCTCCTTC<br>TCTTCSTCMCWSMSAYTWT<br>CTCTNNNNDGAGVDGGGD<br>VGBVATVTTDTBAAGACGT<br>HTTAATAHBABDGAGVGCG<br>ABAAAAAAAGADVTCTADT<br>GGGAGGGAGGAAGAGAAG<br>GAGAGATAGAGAACGDAG<br>GAGGGGYTSWKMRWSKYK<br>SKAMGGAGAGGGAAGGAG<br>GGAGAGAGAGAGGGAATG<br>GGAGAAAGCAAGAGGGAT<br>AGCTACGCATGGAATATTG<br>AAGGCAGAAAGAGATAGA<br>TTGAGAGAAAGATAG	
EcilSat31-115	TACGGGAATGTGCTCCCGT<br>TATGACAAAGGATCAAATG<br>GGTCTGTGTTCCCATTGATC<br>AAAGGGGATGATTCAGAGG<br>CTAGGGTGTGTTCTAGCTT<br>GAATCACAATACGATGTCT	115
EcilSat75-101	CATAGTCAGCAGGAGATCT<br>TATAGGAGAGAACTTATCG<br>GGTCCTATGAGATTCCTGA<br>AGAAGATCAAAGTGACTAA<br>TGAGGAGAGAGCGTACTCG<br>GGTCCT	101
EcilSat119-132	TAGAAAAGAGTGAAAGGG<br>AGTGAGAATACCATGCGGA<br>TTCACATCTCAACCCTAAC<br>GACTCATGGTTAGGGCAAC<br>CATGCGGACTCACGTCCTA<br>ACCAGGGAGACAGGAAGA<br>CCATGCGGATTCACATCCT<br>G	132
EcilSat51-335	TGTGAATGTATATTTACTTT<br>CHCAYWKYAWATGCAMGT<br>TCAAACCGCGTAGTGAATA<br>TTCATTCACAACGAGGTGT<br>GAATATACATTTACAAAAG<br>GGAAGTGAATATTCGTTAC<br>CCTTGTAGATGTATATTCAC<br>ACATCATTGTGAACGAATA<br>TACGTTCACAATGAGGTGT<br>GAATGTGCATCTACAAGGG<br>CGTAGTGAATATACATTCA<br>CAACGAGGTGTGAACGTAC<br>ATTTACAAGGGTGTAAACG<br>TGCAATTACAAAAGGGAAG	335

	CGAATATCCATTCACAATG ATGTGTGAATATACATCTA CAAGGGAATATTCAGACCT CGTTGTGAATG	
EcilSat34-374	TCTGATAGCAACTTTSYKM RWWMSTBCBACVGHTGDV THTTCCDADCAGAVTCDHT CTVTCTGBDAGDACAATTT CTGAKAATCAATAGWCCAC ATGGGACKGCCGGGCGAA ASYAAGACTWAGGGTCCGT TTGGCCCGGCTGTGCTGTC AGAAGAATCTGCTGTCAGA AGAAATTGCTGTCAAATTT TGTTCTTGCTWACTGTTTG GTGTCTGTTTGGAAAACTC AGCTGTGTGACTATTGTCT GCTGWCAGAAGAATCTGCT GTCAGAAGCGGGGCCAAAC GGACCCTAAGTCTTCTTTCT TTCMKGATGRAATTWCTAA WTATTTBATTCAGWAATTT AGGADGDVDTYYTTKWTT YYMKRRMWYWTACTC	374
EconSat36-134	GATTCACATCCTGTCAAAG AAAAGGAAAAGTGGATTG AGAATACCATGCGGGTTCA CATCTCAATCCAAACAACT CGTGGTCAGGGCAACCATG CGGACTCACGTCCTAACCA GAGAGACAGGAAAACCAT GCG	134
EconSat195-120	GACTTAAGCTAGGATATGC CCCGGCCCCTGAGTCCCTA CATTTCCTTCGATCAATGG GCACATACACCCATTTGAT CTTTCGTCGAGACGGGCAC ATACACCCGTAAGACTTCG TAGTGT	120
EconSat233-246	ACTCTCTCTCTCTCTCTGGC CAACAGAGCCATCGATTTC AGCTAGGGCAACACCCAAT AGATAAATGACCATTCTCA TTCTGGTCAACAGAGCCAC CGATTTTGGATCATTGGAA AGGAAGCCAATCTTTGGCT AGGGCATAAAATGAAACA AGGGGAATTTACATAAATA CCACAAAATTTTAACGAAT TCAAGAGAAAGATGAAAA AACACAAAAATAGAAAG	246

	ACGAAAAAAATCCAACAAT<br>C	
EconSat253-126	CATATCTTAAAGTAAATTA<br>AAACTTTGAAAATTAAATT<br>AAGTTACTTAACTTAGGAA<br>AGTTAAGTCACTTAATATT<br>CAAATGAAATTTTAATTAA<br>GATAGATTAATTTGATATC<br>GATTCAAATTAA	126
EconSat1-208	TADGGTTTGGGTCTTGACT<br>AAGCTTAAAGTGTGACCGG<br>ACCCAATGGGTCCCAAACT<br>TGTCTAGTAGGCTTCTAGT<br>GTATGTGTGAAGGTTTGTG<br>AGAAGTTTGGTCCAATTTG<br>GAGATGTTTGACCAAACAC<br>CTTAACTTTGACCTATTTAG<br>GWCAAATTGGCCCCACTCA<br>CACTTTAAGCCTAGTCAAG<br>ACTCAAACCCTAASCAG	208
EconSat2-197	ACCTRYMYACATACACTAG<br>AAGCCTACTAGACAAGTTT<br>GGGTTAGGTTTGGTCAAAT<br>TTTGATGTTTGACCAAACA<br>CCCTAACTTTGACCTATTTR<br>ACCAAATTGGCCCAACTTA<br>GGGTTTGGNTCTTGACTAA<br>GCTCAAAGTGTGACCAGAC<br>CCAATGGTCCCAAACTTGT<br>CTASATAGACTATAAGYCT<br>ACTAGA	197
EconSat3-213	GTAGGTCAAAGTTARGGTG<br>TTTGGTCAAACAWCTCCAA<br>ATTTGACCAAATTAGGYCA<br>AATAGGCCCCACTTAGGGT<br>TTGGATCTTGACTAGGCTA<br>AAGTGTGACCGGACCCAAT<br>GGGTCCCAAACTTGTCTAG<br>TAGGCTTCTAGTGTATGTGT<br>GAAGGTTTGTGAGAAGTTT<br>GGTCCAATTTGGAGATGTT<br>TAACSAAWCASSYMAMMY<br>ATAA	213
EcusSat12-472	GCAAAAAAGGAGTGCAAC<br>ACGAGGACTTCCCAGGAGG<br>TCACCCATCCCAGTACTAC<br>TCTCGCCCAAGCACACTTA<br>ACTGCGGAGTTCTGATGGG<br>ATCCGGTGCACTAGTGCTA<br>GTATGGTCACACCCGCATG<br>AATTTGAGCGTGCAATATA<br>CTTGTATGAAACTTCCCCG	472

	AGCGAAGGAGCCGCACCAC<br>AGCCGAATCATGCACCGTG<br>GGCGGGGCCGAGGTGGCCT<br>TCGGCTACTCGTCCCGTCC<br>GCGGGCTCATCGCGGCGTT<br>TCCGAAATGGGGTCATAAT<br>AGCAAAATGGAGCCCTGTG<br>CGAGGAGGTGCGTCGATTT<br>TCATACCACAACTGAACGG<br>GATGCACGGATCCAGCGTG<br>ATGCGCCTTGGCTTTGGAT<br>CCTCGGATGGCATGCTCGA<br>GGCCGACCTCCGGGATGCG<br>GTGTGCATGATAACTTTCG<br>ACGGGCTCGGGAACAGATA<br>GCGCGAGACGAACCGCG	
EcusSat164-120	GCCTCTAAGTCCCTACATTT<br>CCTTCGATCAATGGGCACA<br>CACACCCATTTGATCTTTCG<br>TCAAGACGGGCACACACAC<br>CCGTAAGACTTCGTAGTGT<br>GACTTAAGCTAGGATATGC<br>CCTA	120
EcusSat71-225	TCAACTCAGGGCACTACTC<br>TGATCCTATGAGAATCCTA<br>AAAAGGGGCGACTGGTTGG<br>GGGGTATCCACTAGAGGCT<br>CATACCGGTCCAATCAGGG<br>CTAGGACCAACAAGGAAA<br>GTGTCCACTAAGGGTCTAC<br>ACTAACCTTGGGTCAAAAG<br>GTGACTAATGAGGAGGGAG<br>ATCCATCGAGGGTTTACTC<br>TGATCCTAATAGTCAGGGA<br>TCCGTAGGGGAGAGGTG	225
EcusSat96-132	GCATGGTTGCCCTAACCAT<br>GAGTCGTTAGGGTTGAGAT<br>GTGAATCCGCATGGTATTC<br>TCACTCCCTTTCACTCTTTT<br>CCACAGGATGTGAATCCGC<br>ATGGTTTTCCTGTCTCCCTG<br>GTTAGGACGTAAGTCC	132
EcusSat5-232	CCCACTTAGGGTTTGGGTC<br>TTGACTAGGCTCAAAGTGT<br>GACCAGACCCAATGGGTCC<br>CAAACTTGTCTAATAGGCT<br>TCTAATATGTSAAAGTTAG<br>GGTGTTTGGTCAAATATCT<br>CGAAATTTGACCAAACTTC<br>CCACAAACCTTCACACATA<br>ACTAGAAGCCTATAGACAA<br>GTTTGGGACCCATTGGGTC	232

	TGGTCACACTTTGAGCCTA<br>GTCAAGACCCAAACCCTAA<br>GTGG	
EcusSat20-260	GGRTCAAACATCTCCAAAT<br>TGGACCAAACTTCCCAYAA<br>ACSTTCACNTAYATANCTA<br>GAAGCMTRTTAGACAARTT<br>TGGGACYCTTTGACTAAAC<br>ACCCTAACTTTGACCTATTT<br>AGGTCAATTGGGCCCCACT<br>TAGGGTTTTACTCTTGACTA<br>GGCTCAAAGGGTGACTAGA<br>CCCAATGGGTCCCAAACTT<br>GTCTAATAGGCTTCTAGTG<br>TATGTGTGAAGGTTTGTGG<br>GAAGTTTGGTCCAATTTGG<br>AGTTGTTTGAC	260
EdifSat97-156	GAGGTCCTAGGTTGACCAG<br>GTCAACCCTGGTCAACCTA<br>CTTCCTTCACAAACCCTAGT<br>TTTGACCTAAACCCTAATT<br>AGGTCATAACTAGGGTTCC<br>ATACACCCACATGTCACCA<br>AACTTGGCATGGGGTGTAC<br>ACATACCCTAGTGGACCCA<br>TTT	156
EdifSat1-290	ATBVDHSTTACMASATGTY<br>BGMCKAGCCSAYGCCMMM<br>CCCMYCCGACWTYKCCYY<br>CTCGATTCCCCCCTTCCTCT<br>CTCTTCCCCTCCCTCCGTYT<br>CCTCCGACTACACCGAAGA<br>AGAAGAAGAAGAAAAGAA<br>GAAGAAGAAGAAGAAGAA<br>GAAGAGAAGAAGAAGAGA<br>GATCGGAAGAGCACACGTC<br>TGAACTCCAGTCACCGGCT<br>ATGATCTCGTATGCCGTCTT<br>CTGCTTGAAAAAAYTGGRG<br>GGTGRKDGGGGARATTKDA<br>KSGGKWKRGCCTCCAATCA<br>AACCAGT	290
EdifSat2-326	GCAGAGAGCTGTTCSSWW<br>MMMYCTDTTCTGYCSACTG<br>YAGCCCTACMCAWCCCTCT<br>TCCCTTTTCTCTCCCCCTCT<br>MYKCCTTTCCCCTCACTTCT<br>CTTCCCTCTTCCTCCCCCCC<br>TCTTGCCATAAGAAGAAGA<br>AGAAGAAGAGAAGAAGAA<br>GAAGAAGAAGAAGAAGAA<br>GAAGAAGAAGAAGAAGAT	326

	CGGAAGAGCGTCGTGTAGG<br>GAAAGAGTGTTAAGATTAG<br>TGTAGATCTCGGTGGTCGC<br>CGTATCATTAAAAAAAACC<br>CAATTTTHCCCTCWTGANN<br>GCNHTGGCCGTTCCTTCTC<br>GTCCCCACTGAAACCGATG<br>GCAC	
EdifSat7-324	AAAGACGAGGGNNNNNNC<br>NCNACGGNGNGSTMGKGW<br>RVDGGAAKGRAGRGGRAG<br>ARAAGGAATTGTWCKATTA<br>SGATGAGAAAKAAAGGWA<br>TACTTYATATATAAGTTTCT<br>TCTTCTTCTTTCTTCTTCTCC<br>TTCTTCTTCTTCTTCTTCTTC<br>TTCTTCTTCTTCTTCTTCTTC<br>TTCTTCTTCTTCTAGATCGG<br>AAGAGCGTCGTGTAGGGAA<br>AGAGTGTTAAGATTAGTGT<br>AGATCTCGGTGGTCGCCGT<br>ATCATTRGARRAGCAGACA<br>TAKACTTCDGKAAMCYTGC<br>CTCCGNCYTTGTCTTHHBG<br>YKYKCWYTTCCTCTAT	324
EdifSat8-248	ATTACAATACTAGGCCTTA<br>ATTCATAAAGGATACAWRS<br>RARGRARAVGVACWTCCG<br>AKAAWTATTAACATMACG<br>RWMCCTTAACACTCTTCCT<br>CGTCATYTTCTTCTTCTGGA<br>ATCTTCTTCTTCTTCTTCTTC<br>TCTTCTTCTTCTTCTTCTTCT<br>TCTTCTTCTTCTTCTTCTTCT<br>TCAGATCGGAAGAGCACAC<br>GTCTGAACTCCAGTCACCG<br>GCTATGATCTCGTATGCCG<br>TCTTCTGCTTGAAWA	248
EdifSat116-214	GAATWKSCGWCGSAAWKT<br>CCGNYCGGTGAWGCCTGG<br>ATAAACCTAGGTCANCGAA<br>CCCBAAACCCATTTCTCTCT<br>CGCTCGGCTCGGCTCTCTCC<br>CCTCACCCGAVGCTTTTNCT<br>CTNCTCTCCCTCCTCTCTCA<br>CGATCGGAAGCCCCGGCCG<br>GCCGTNACGCAATCGCCGC<br>CGCCGTCGCCGCCGACGTC<br>GCCGCCGCCGTSGRYWYGG<br>GGR	214
EdifSat4-529	AGGAATCGGGAGGGCGAC<br>GTCGCGTCGCCTCCCTATTC	529

	CCGGATTCCYCCTHTHCAA<br>TTBGATNAGTTWAKGAGKT<br>GAARGBGWTAAVADCGAA<br>GAMTATTTAGGAGGAAAC<br>GTGARTRAAWGAGMAAGA<br>GAGAASTAGMAGCGAATTC<br>GCCGGAGTACAAGAAAGA<br>AGAAAAAACTTCTCAGANA<br>AAACTCCTTCTTCTTCTCTT<br>CTTCTTCTTCTTCTTCTTCTT<br>TTCCTTCTTCTTCTTCTTCTT<br>TTCTTCTCCTTTTCAGAAGA<br>AGAAGAAGAAGAAGAAGA<br>AGAAAAGAAGAAGAAGAA<br>GAAGAAGAAGAAGAAGAA<br>GAAGAAGAAGAAKAAGAA<br>GAAGAATAAGTAGAAGAA<br>TAATAATAANAAAGAAGG<br>AAAAAAGAAAAAAAGAAA<br>AGATKAAKCAGAAGAAGA<br>AGANGANGAAGAAGAAGA<br>AGAAGAAGAAGAAGAAGA<br>AGAAGAAGAAGAAGAAGN<br>AGNNNGNNNNNNNNNNTC<br>TTCTYCTTCTTCTTCYDTDH<br>CBTHCTBHTBDAVDDVDHH<br>HGGDHT	
EdifSat5-441	CCGACACCCTCKRKYCGAT<br>CGCACGAGSCCCVHCTCGC<br>CCCCYCCSCMCCCCGCCAC<br>CCCCRCCCCCCCCTCCCCCC<br>CCCCCCCMCCCCCCCCCCC<br>CCCCCCCCCCCCCCCCCCCC<br>CCCCCCCCCCCCCCCCCCCC<br>TTCCCCCCKCTACTGTTCAC<br>CCGAAANAGAAAGAGGAA<br>GAGGAAGACGGAGAAGAA<br>GAAGAAGAAGAAGAAGAT<br>TCGCCGGAGGAAGAGGTAA<br>CTCTCCTTCTCCTTCTTCTT<br>CTTCTTCTTCTTCCTTCTTCT<br>TCTTCTTCTTCTCTTTTTCCT<br>TTTTCTTTTCTCGGGTGAAC<br>AGTAGAGGGGGGAAGGGG<br>AGGGGGGGGGGGGGGGGS<br>GGTGGGSGVGGGGGGGGW<br>GGGGGGTGAGCGRGGGGG<br>AGGGCTCTSCRWTTCYTGC<br>GAGKTSTGNGCKCTTGGHG<br>CAGGTCGGACGGAGGAGC<br>AC	441

EdifSat6-478	GCTCTCGGTYCMSHCCTVG<br>TCTCCCNACSTCCCCYCCBY<br>CCYATCHGATCGCCGMACR<br>AGCCGAGCGGACABCSCYC<br>CGCTSCCCCTCCCCCCMTG<br>CCCCCTCSYCCTCCCCYCCC<br>CCCCCCTCCCWCCYCCCCS<br>CCCCCCCCCCCCCCCCCCCC<br>CGCCCCYCCCCCCCTCTGCT<br>SCYCGCCACCCAGCCGAAC<br>CCCCCTCTTTACTGTTCATC<br>CGAGAACAGTGAAGAAGA<br>AGAAGAAGAAGAAGAAGA<br>AGAAGAAGAAGAAGGAGC<br>GAAGGAGAGAGGGAAGTT<br>TCCCTTTCTCCTTCTTCTTCT<br>TCTTCTTCTTCTTCTTCTTCT<br>TCTTCTTCTTCTTCTCCCTTC<br>TTCTCGGATGAACAGTGAA<br>AGGGGGGGKGGGGGGGGG<br>GTATGGACVRGSAGGAGGA<br>GRGTGGCGCVCKGCMGGA<br>GKMGAGHGVBHTCTGHGG<br>KKSGRKMASARYRGRKCAG<br>GCGAGCAGCAGGGTCGGT	478
EdifSat45-294	CCATCCGCACGTTCCCCAA<br>GCTTCTTCAGAARGCAGCG<br>AGCCTCGCTGAAGGCKCTT<br>CTCAGCTGGGTTTTCTNAA<br>GGATATGGCGACTAGCGAC<br>CCTAAGAAGAACCCGCCGC<br>CTCAGAACAAGAAGAAAG<br>GAGGCCAGATTACCGCAGT<br>GGGCAAGGGGAAGCAGCA<br>GCAGGAGGACCCAGAGCTA<br>GTCGTGCCAGGACCWAGY<br>CTCCCTCCTCCCAGNCAGC<br>AGCAGCCTCGACCGCCCAG<br>ACCTCAGCAGCAGTACAGG<br>CCGCCNYAGTACCAGYAGC<br>CGCAGCAGGGCC	294
EdifSat16-342	KWRWRMWSWWSMNNNNN<br>NNTTNTTNNNNTTTATAMK<br>AMGTMGTATAAAGAAGAA<br>GAAGAAGAAGAAGAATTA<br>GTATAAAKAAGAAGAATA<br>ATTATTATAAAGAAGAAGA<br>AGAAGTAGTATAAAGAAG<br>AAGAAGAAGTAGTATAAA<br>GAAGAAGAAGAAGTTTATT<br>ATAAAGAAGAAAGAGTAT<br>AAAGAAGAAGAAGAAGTA	342

	GTATAAAGAAGAAGAAGA<br>AGTAGTATAAAGAAGAAG<br>AAGAAGAAGTAGTATAAA<br>GAAGAAGAAGAAGTAGTA<br>TAAAGAAGAAGAAGTAGA<br>AGAAGAAAGAAGAAGAAG<br>AAGAAGAAGAAGAAGAAG<br>TARNADAAAGAAGTAT	
EgasSat152-202	CCTCCTATTAAGATCTCCCC<br>CTGACTATTAGGATCAGAG<br>TAAACCCTTGGATCCCTCCT<br>CATTAGTCACCTTTCGACCC<br>AAGGTTAGTGTAGGCCCTT<br>AGTGGACACTTTCCTTTGTT<br>GGTCTTAGCCCTGGTTGGA<br>TCGGTATGTGGATACCCCC<br>CAACCAGTCGCCCCCTTTT<br>AGGACTCTCATAGGATCAG<br>AGTACATC	202
EgasSat182-117	TGATCCTTTGTCGAGACGG<br>GAACACATACCCGTAAGAC<br>ATTGTAGGGTGATTCAAGC<br>TAGAACACACACTAGCCTC<br>TGAATCCCTACACCTTTGAT<br>CAATGGGAACACAAACCCA<br>TT	117
EgasSat221-133	CCCCCGTCAGGATGTGAGT<br>CCCCGAGCTTTCCTGATTGT<br>GGGCTGCGCAGGAACGAG<br>ATGTGAGTCCCCGCGCTTT<br>CTCGTCTCCTTTACTTTTTC<br>TTAGGAACAAGATATGAGT<br>CCCCGTGCTTTCTTGTTC	133
EgasSat239-207	GTCGGAAAATCAGGGGGGT<br>TTGTATCCTTCATAGTTTAT<br>GAAGGGGCCCTGAAAGGTA<br>AGGTGATCAAGTCCGCGTG<br>CCCAGGACTTGATACCAAA<br>AGAAATTTCCTAGATCCGC<br>GTAACCAGGATCATCGGAA<br>AAAGGGAGGTTATCAAGTT<br>CGCGTATCCAGAACTTGAT<br>ACCGAAAGGGGTCCTAGAT<br>CCACGTGCCCAGGATC	207
EgasSat258-100	AGATCAAAATGACTAATGA<br>GGAGAGAACTTATCGGGTC<br>CTCATAGTCAGCAGGAGAT<br>CTTATAGGAGAGAACTTAT<br>CGGGTCCTATGAGATTCCT<br>GAAGA	100
EgasSat43-380	AAATATGAVATVCTTTHAG<br>DTAAANATMKATWHTKCT	380

	THGAWAAGWATMWBGAC<br>CAAACGGAGACTTAGGGTC<br>WGTTTGGCCCAGCTTCTTC<br>TGTCAGCTGWCAGCAACTT<br>CTTCTGTCAGCAACTTTTTY<br>CATCACTCACACAGCTGTC<br>ACACAGCTGAGTTTTCCAA<br>ACAGAAACAAGAACAATTT<br>CWGACAGCAGCTTTTCCTG<br>ACAGCAGNTGACAGCAGA<br>GCTGGGCCAAACTGAGCCT<br>AAGTCTTWRTTWGGCVMR<br>RCTGTGCAAGCAGAWTCAT<br>CTGYCAGMAGKAAAAKCT<br>GTCARCAGCTTTTDBHATC<br>DBTBCCAHABBBGDCAHTV<br>AGCTGTVTGTTHGVADAAM<br>WCMRMKRWSWRWKTSAK<br>RSAGAAA	
EgasSat263-88	AGGTTCCTAGATCCGCTTCT<br>CCAGGATCATAGGAAAAAT<br>CAGCTGAAAGGTAGTGAAC<br>AAGTCCGCTTCTCCAGGAC<br>TTGACACTAGA	88
EjueSat37-225	AGTAGTGCCCTGAGTTGAC<br>ACCTCTCCCCTACGGATCC<br>CTGACTATTAGGATCAGAG<br>TAAACCCTCGATGGATCTC<br>CCTCCTCATTAGTCACCTTT<br>TGACCCAAGGTTAGTGTAG<br>ACCCTTAGTGGACACTTTC<br>CTTGTTGGTCCTAGCCCTGA<br>TTGGACCGGTATGAGCCTC<br>TAGTGGATACCCCCCAACC<br>AGTCGCCCCTTTTTAGGATT<br>CTCATAGGATCAG	225
EjueSat89-162	TTCCTTTTTGCGCTCTACAT<br>CATTTTAAGGAAAATAATG<br>GTACACATGTGTTGGACTT<br>GGACAAATTTACAAAAAA<br>GCAGATTATCTGCTTTGATT<br>GAATGTAGAGCGCAAAACT<br>GTTATATGTCGCTGGTGTA<br>ATTTTCTATTATAAAAATAC<br>GTCTTGT	162
EjueSat112-176	TTCGCTTATAGGAGGCATA<br>TGTTCAGAAATTGTCAATTT<br>TGACTGTGCCAGACGGCAC<br>AACAAACGATTTTGAACAT<br>TGGCATGAATAACTGACAT<br>GTCTAGCTTCCCCCTGGAC<br>CAAAAATTGCTAATGCACA	176

	ACGAGCCGTTTGGACACAG<br>TGGACACTTTTCTGACGCA<br>TATT	
EjueSat143-101	CTATAATTCACGTATAAAT<br>GCTTCACAATCGCAATATC<br>TGGTCGTTTTCGATCATTTT<br>AGCGTATTTCCAGCCGTTT<br>AGAGCGTTTACATTGTCAT<br>TTCGC	101
EjueSat184-153	CATCAGAATCGAGCGTTTT<br>AACGTCCGAAACGCTCAAA<br>TATGCTATTTTTAGGCAATA<br>AAACGAGTGCGTTTCTCTA<br>ATATAACTTGAATCAACTG<br>CAAAATACTTACTTCTTGA<br>GTAATTTTGGTCGAAAATT<br>ACATATCTGCTATCAAAAG	153
EjueSat347-488	AGATGGTCAGTTGCTCAAT<br>AGAACATATTCCACTCAAG<br>TTATGAGGCAAAAACGTTT<br>TTTTTCCTAGTTTTTTGACA<br>ATATTTCGGAAACTATAAA<br>TGCTACAGTGACCATTCTA<br>GCAGCATTCGAAAGGTCTT<br>TTTGTCCTCTTTAATTTGAT<br>ATATAACAAGTTACCCTTA<br>TCTATTCAAATGCCAGAGA<br>AAATCGCAATTTAGTAAAT<br>TTTCAAAAAGTGCACTTTT<br>ACATAGGATCTTTCTTATTT<br>ACAGTCCGATTGGGGTAAT<br>TCTGGTCTATATCGATGCA<br>GTTTGACATCCTGATTCCA<br>AAAATGACCTTAAAATTGC<br>CGTAGCTCCAGTGGTTCGG<br>GAGAAAAAGCCGAAAAAC<br>GCATTTTGAAAAAAATTCA<br>AAGGTAGTTACCTTACAAC<br>ATTTTTTACCCATCGAAAA<br>AATTTTTTTCTCTCAAAATG<br>AGATAAAATCATGCTGAAT<br>CGATAGAGAATTTTCTGTTT<br>ATTCTATCT	488
EjueSat69-134	GTTGCCCTAACCATGAGTC<br>GTTAGGGTTGAGATGTGAA<br>TCCGCATGGTATTCTCACTC<br>CCTCTCACTCAATTCTCTAC<br>AGGATGTGAATCCGCATGG<br>TTTTCCTGTCTCCCTGGTTA<br>GGACGTGAGTCCGCATG	134
EjueSat14-233	TGGACCAAACTTCCCACAA<br>ACCTTCACACATTTRTGGG	233

	AAGTTTAGTCAAATTTGGA<br>GCTGTTTGACTAAACACCC<br>TAACTTTGACCTATTTAGGT<br>CAATTGGGCCCCACTTAGG<br>GTTTTACTCTTGACTAGGCT<br>CAAAGGGTGACCAGACCCA<br>ATGGGTCCCAAACTTGTCT<br>AATAGGCTTCTAGTGTATG<br>TGTGAAGGTTTGTGGGAAG<br>TTTGGTCCAATTBGAAGWA<br>CAC	
EjueSat6-199	ATAMATTGGGTCTGGTCAC<br>ACTTTGAGCCTAGTCAAGA<br>CCCAAACTCTAATWGGCTT<br>CTAGTATATGTGTSAAAGTT<br>TGTGGGATGTTTGGTCAAA<br>TTTGGAGATGTTTGACCAA<br>ACACCCTAACTTTGACCTA<br>TTTAGGTCAACTAGGCCCC<br>ACTTAGGGTTTGGTCTTGA<br>CTAGGCTCAAAGTGTGAYC<br>AGACCTAA	199
ElacSat73-132	GGGAGACAGGAAGACCAT<br>GCGGATTCACATCCTGTGG<br>AAAAGAGTGAAAGGGAGT<br>GAGAATACCATGCGGATTC<br>ACATCTCAACCCTAACGAC<br>TCATGGTTAGGGCAACCAT<br>GCGGACTCACGTCCTAACC<br>A	132
ElacSat98-115	CAATGGGAGCACAAACCCA<br>TCTGATCCTTTGTCAAAAC<br>GGGAACACACCCCCGTAAG<br>ACATCGTATTGTGATTCAA<br>GCTAGAACACACCCTAGCC<br>TCTGAATCCTCCTCTTTGAT	115
ElacSat107-193	CTCTTTTAGGACTCTCATAG<br>GATCAGAGTACATCCCTCC<br>TATAAGATCTCCCCTTGACT<br>ATTAGGATCAGAACACATT<br>CTCTCCTCATTAGTCACCTT<br>TCGACCCAAGGTTAGTGTA<br>GACCCTTAGTGGACACTTT<br>CCCTTGACTGGTCTTAGCCC<br>TGTTTGGATCGGTACAATA<br>CCCTCCAACCAGCCGCAT	193
ElacSat300-119	ACTACACTTCAGGTTGGTC<br>TTTCTGTCCAGCTACAGATT<br>GACCCCTTGTCTGGTTGTTC<br>GAAGATTAGGCTGACTTCT<br>ACCGGTCACCACCTATTCC	119

	TAGATCAGGCATTGGACAC<br>CTT	
ElacSat309-120	CGCCCATTGATCGAAGGAA<br>ATGTAGGGACTCAGAGGCT<br>AGGGCATATCCTAGCTTAA<br>GTCACACTACGAAGTCTTA<br>CGGGTGTATGTGCCCGTCT<br>CGACGAAAGATCAAATGGG<br>TGTATG	120
ElacSat46-391	TACCCCACTACTTAGGGTC<br>AGTTTGGCCCGGCTGTGCT<br>GACAGAAGAAGCTGCTGTC<br>TGAAATTGTTCTTGTTTCTG<br>TTTGGAAAACTCAGCTGTG<br>TGAGTGATGGAAAARGYRG<br>MTKACGGNWTTTCYGGAG<br>GTWCTTTTGTMTGARAAGC<br>CAGGCCAAACRGAGACTTA<br>GGGTCAGTTTGGCCCGGCT<br>GTGCTGTCAGAAGAAGCTG<br>CTGTCAGGAAAAGCTGCTG<br>TCTGAAATTGTTCTTGTTTT<br>GTTTGGAAAACTCACAGCT<br>GTGTGAGTGATGGAAAAAG<br>TTGCTGTCAGAAGAAGYTG<br>CTGTCAGCTGKCAGAAAAG<br>CTGGGCCAAACTGACCCTA<br>AGTCTGAGTTTGGTGCGGC<br>TTCARARGGMARCGRATGG<br>YMSGWTTGAC	391
ElacSat104-354	MKSKMKTKSKMSSSSWYKS<br>WSKSNNNNNNNNNNNNAG<br>GGAGAGAGAGTGAGMRAG<br>RGRGAGAGAGAGAGAGAG<br>AGAGAGAGAGAGAGAGAG<br>AGAGANNGANNNNCTCTCY<br>CTCTCTCTCTCTCYACCTCT<br>CWCTCTCACTCACTCTCTC<br>CCTCCCTCCCTCTCCCTCTA<br>GTTTTTAAAAGAGGAGGTT<br>GGCCGGTACAAAGTCTATT<br>AGAATAAAAGGTGCAACGT<br>AGAAGAGGTCTCTCTCTCT<br>CTCTCTCTCTCTCTCTCTCT<br>CTCTCTCTCTCYCTCTCTCT<br>CTCTCTCYCTCTCTCCCTCC<br>CTCTCTCTCTYTYYATCCCC<br>TCCCTCTCCCTCTCTCCCCC<br>CACTCCCTC	354
ElonSat208-119	TGATCGGTCGAAATCAGCC<br>TATTCTTCGAGCAACTAGA<br>CAAGGGGTCAATCTGTAGC	119

	TGGACAGAAAGACCAACCT<br>GAAGAGTAGTAAGGTGTCC<br>AATGCCTAGTCTAGGAATA<br>GGTGG	
ElonSat264-150	CTCCCTACATTAACAACCC<br>ACCAACCCCTCCTCCTTCTC<br>CATTATCACCCTCTCTTTCT<br>CCTTCCCCATCGTCTCCTCC<br>CTACATTAACAACCCACCG<br>ACCCCTCCTCCTTCTCCATT<br>GTCACCCTCTCTTTCTCCTT<br>CCCCATCATCTC	150
ElonSat291-166	GGTTAATTCTAACCCAAGG<br>ATAGGGACCTAAAGCTCTC<br>CCTAAGTTAGTGATGCCAT<br>TATACTATTTAATGATTAAT<br>TAAATCCTAAGGATTTTAC<br>TTAAGTATTTAACTAATCAT<br>TAGAGGAAAGGCTTGATAT<br>AATTTAGGGAGAGGGTGAA<br>GGTGATTCCAAG	166
ElonSat327-156	GTTTCAGTGCAGGTACAGT<br>TTACTTCAGGAGCTGTCGA<br>AGACACACGACACGAGTAG<br>CTACGCTGCGCGCTGGCGA<br>TCTCGTGTCACCCGCACTGT<br>GCACCCACGCCTGGGTTGC<br>TGAGCTGCGCGCTGGCGAT<br>TCCGGGCAACGCTAACGAT<br>TGT	156
ElonSat421-462	CAAAAACCACCGTTCCACT<br>TCATCTCCTTCTTCTGCTTC<br>CTCTCGTCATCAGCTCCCTC<br>CTTCTCCCAGCCTGCTCCCT<br>CCTTTGTATTCTTTCCACCA<br>CGACTACGCTCTCCCCTCTC<br>TTTCTTCTCGCTAACATCTT<br>CTTTATCCTTCTTCTTGCTA<br>TCACCAACCTCTTTCTTCTT<br>GCCATTGCCACCAAAACCA<br>CCCTCATCCTCTTCCTCATT<br>CTTCTTCTCACCACTGCCCT<br>CCATCTCATTCGTCTTCTTC<br>TTGTAGCTGCCTCCCCATTC<br>CTTATTCCCACGATCGCCG<br>CCTCTCTCCTCCTTGTGGAA<br>CACATCTCTCACTTTTTTCT<br>TCTCGTCACGACCATTGCTT<br>CTCTTCTCTTCCTTCTCTCC<br>ACCGCCACCGCCTACCTCC<br>TCTTTTTTCTTGCGATTGCC<br>ACCACCCTCCTCCTTCCCGC	462

	GGCCACTACCTCCCTCCTCT<br>TCCAAC	
ElonSat110-282	TCACAAGCATGAGGAGCAC<br>GMKKWRCTCYAGCATCAG<br>RAATGARADGAAGGGAAA<br>CTTAGGCTGAGTTTGGCCC<br>AGCTTCTKMAAKCAGCTGT<br>CAGCAACTTTTTCTGACAG<br>CWACTTTSTCCATCACTCA<br>CACAGCTGAGTTTTYCAAA<br>CAGAAANCAAGAACAATTT<br>CAGACAGCAGCTTTTNCTG<br>NCAGCACAGCCGGGCCAAA<br>CTGACCCTAAGTCTBMAAK<br>WGWRGKWTCTKCCAKTTA<br>ATWWCTBADARMKRKWG<br>MSRTGCTAGGTCAATTATC<br>TG	282
EmahSat69-103	ATAAGATTAAGGATTCATA<br>GGCGGTGCCCAGAGGAATC<br>CAAAAGAAGTGATTGGTTG<br>GAGCTGCCTTGCTTCAGGT<br>CTTACATACTCCTAACCAA<br>CACTTAAG	103
EmahSat81-134	GGAAAACCATACGGGTTCA<br>CATCCTAACGGGGGGAAAC<br>AGGAAAACCATGCGGATTC<br>ACATCCTGATCCAAAAAGT<br>AAAAGGGAACGAGAAAAC<br>CATGCGGGTTCACATCTCG<br>GCCCTACGCAGCCCACAAT<br>TA	134
EmahSat267-673	TGTCCCCCACCGCAATGGC<br>CACGACGTCGGCCTCTACC<br>TTGACGCCGCCTGCTCCCTC<br>GAAGACCGCGCCTGCGCAT<br>GCTACGGGGCTCCCCTCTC<br>CATTTCCTCTGACGAATTTG<br>TCGAATCCCTCGTCCTGGA<br>CTCCATATTTGCCCTCGAAC<br>TTTTGCTCGGAATTTGTTGC<br>GACTTCCAAAAACTCGGCT<br>ACGGCACCAACGACCTCGT<br>CTTCTTTAACCGCAACGAG<br>GTCACCATGTTGACGATCG<br>TGCGCGATATGATCTTGCT<br>CGAGAATCAGATCCCTCTC<br>TTTGTACTTGACCGCATCCT<br>TGCCCTCCAGCAGATTGAC<br>CACATCCTTGTCCTCCTGCA<br>CGGCCTCGAACTTCCTACC<br>TCAGGCCTTGTCGCTCTCCT	673

	CGCCTTCGATTCCTTCTGCA<br>ACCTTATTAGGCTCGCTGA<br>CGACTCCCCTTTCGACTATT<br>CCTCGAATCCTATTTCCGAC<br>GCTGGCACCAGTGAGGCCC<br>TCCATTGCCTCGATGTCTTA<br>CGCCGCAGCCTCCTCCTCCT<br>CCTGGGCGAACCAATCCAT<br>CTACCGCGTCCCCAAAGCC<br>CTCCGTGACGTTGACGAGA<br>TCGCCTACAACCCTCAGAC<br>CGTCTCCCTCGGTCCCTACC<br>ACCGCGGCTGCTTCCGCGA<br>CATGGATCACCACAAATGG<br>CGCGCCCTCCACCA	
EmahSat72-108	AGGTCTGATAGGAGAGAAT<br>GGGACTGATCCTATAAGAG<br>TCCTAATAAGGTGACTAAT<br>GAAGGAGGATGTCCTCTAT<br>GGGCTCATCCTGCTTCAAA<br>ATAGTCAAAGGGG	108
EmahSat80-164	CCAGTCATCTATTTTAGATT<br>CATATAGGATTGGAGCTAG<br>CCTTAAGTGGCACCTCTCCT<br>ATGAATCTTTTACTATTTGA<br>GCATCGGGAGCAAGCCTAT<br>AGCGGCACCCTTTCTCAAT<br>AGTCATTCAATCAAGGTTG<br>GAGCAAGCCTTAAGTTGGC<br>ACCTTCCAA	164
EmahSat90-217	TAATCTGAGAGTTCAAACT<br>AAATAGATGATTTAGGGTT<br>TATGATTCTAAGAGGGGTT<br>TGGAGGTCCCTGCCTTAGA<br>GTCATGAAGACTCCTTGGG<br>TCACCTTCATTCCCAACCTT<br>AGATTAATAAATCCTTTTAT<br>CACGAAATTAATTTAGAAA<br>TTTAATAATTCCCTTTAGAG<br>ATTTAAAATCTTGATTAATC<br>AGGACTTTAAATAAATCTT<br>TTGA	217
EmahSat99-139	TCAAGAGATTTACATCCTG<br>GCCAAATAGGAGGGAGCA<br>GGAATACTGTCAGGAGATT<br>TACATCCTGCTCCGAAAAG<br>ATCAAGGTTAGGAATACTG<br>TCAAGAGATTCACATCCTA<br>ACCAAGGGATAGGCACAG<br>GAATACTG	139
EmahSat124-109	GTTGGTTGGAGGGTGACTA<br>TCTAGAAGTCTAATCCGGT	109

	CTAACCAAGGCTAATGGAG GTCTTATAGGAGAGAGATA CTCGGGTCCTATGAGAGTC CTAAAAGATATGCG	
EmahSat177-124	CGTCTTACGGGTATGTGTG CCCGCCTCGACGAAAGATC AAATGGGTTTGTGTTCCCA TTGATCAAAAGAAATGTAG GGGCTTAGAGGCTAGGGTG TGTGTTCCCTAGCTTAAGCC GCATTGCAA	124
EmahSat112-90	TTCTTGGGGCACGTTCACA CCTCATTGTGAACGGAATT AACCCACCTGTAGGGCACT TTCACACCTCATTGTGAAA GGAATTAACCTACC	90
EmahSat25-398	TTTGACCCAGCTTTTTCTGA CAGCTGGCWSMRRMWWM KTYKKWSWSYKACHTTBTC CHTBATDAHTDAATHTTBC AVACAGVAAAATTHAVCH ADCAVAAACAADBACAAA CAAGAACTHTTTCTGACAG CTSSTKTTTTYMASAGCACA GMWGAGCCAAACCRAGAC TAAGGGTCAGTTTGGCCCA GCTTATCCNCAGCTGTCAG CAGCTTCTTCTGTCAGCAA NNCTTTTTCCATCACTCACC TGACAGCTGAGTTTTCCAA ACAAAACAAGAACAATTTC AGACAGCAGCTTYTCCTGA CAGCGCACAGCACAGCCGG GCCAAACTGAGCCTAAGTC TTGGTGTGKTTCATMTTAT AATKGAACRBTCATTMAAC WAGCAGCTAHTTTTTSMWT T	398
EmauSat13-371	GCATGAATATGAGCGTGCA ATATACTTGTACGAAACTT CCCCGAGCGAAGGGGCAGC ACCACAGCCGAATCATGCA CCGTGGGCGGGGCCGAGGT GGCCTTCGGCTACTCGTCC CGTCTGCGGGCTCGTCTCG GCGTTTCTGAAATGGGGTC ATAATAGCAAAATGGAGCC CTGTGCGAGGAGGTGCGTC GATTTTCATACCACAACTG AACGGGATGCACGGATCCA GCGTGATGCGCCTTGGCTT TGGATCCTCGGATGGCATG	371

	CTCGAGGCCGACCTCCGGG<br>ATGCGGTGTGCATGATAAC<br>TTTTGACGGGCTCGAGAAA<br>AGAGTGCATGACAAGATCC<br>GGTGCACTAGTGCTGGTAT<br>GGTCGCACCC	
EmauSat84-225	TGACTAATGAGGAGGGAGA<br>TCCATCGAGGGTTTACTCT<br>GATCCTAATAGTCAGGGAT<br>CCGTAGGGGAGAGGTGTCA<br>ACTCAGGGCACTACTCTGA<br>TCCTATGAGAATCCTAAAA<br>AGGGGCGACTGGTTGGGGG<br>GTATCCACTAGAGGCTCAT<br>ACCGGTCCAATCAGGGCTA<br>GGACCAACAAGGAAAGTGT<br>CCACTAAGGGTCTACACTA<br>ACCTTGGGTCAAAAGG	225
EmauSat108-132	AGTCATGGTTAGGGCAACC<br>ATGCGGACTTACGTCCTAA<br>CCAGGGAGACAGGAAAAC<br>CATGCGGATTTACATCCTG<br>TAGAAAAGAGTGAAAGGG<br>AGTGAGAATACCATGCGGA<br>TTCACATCTCAACCCTAAC<br>G	132
EmauSat176-273	CGGGCAATGGCCTCTCGGC<br>ACGATCTTGTTGTCACCGC<br>CGCTGTATGACCGCTTCTG<br>GTTCTCCCTCTCCCTCTGTT<br>AGGGTTTCTACAAGGAAAA<br>TGCATTTTGGGTTTAAGAA<br>TAAACGGGTTACGGGTCCC<br>AAGAATAAACGGGTTTAAG<br>AATGATACTTCTTGCTCGG<br>CGCATCCGCCGCTATGGGA<br>TTGAAGGAGAGGTCGGAGA<br>CCCTCTCGGTTGCCTCGTCA<br>GCTGCATCGGCCATTCGGA<br>GTCGATAATGAATAGGGAA<br>GGAGA	273
EmauSat366-133	AAAGTAAAGGAGACGAGA<br>AAGCGCGGGGACTCACATC<br>TCGTTCCTGCGCAGCCCAC<br>AATCAGGAAAGCTCGGGGA<br>CTCACATCCTGACGGGGGG<br>AACAAGAAAGCACGGGGA<br>CTCATATCTTGTTCCTAAGA<br>A	133
EmauSat443-140	GTTGACTATGAGGACCCGA<br>GTACGCTCTCTCCTCATTAG<br>TCATTTTAGCCCTGCCTGGA	140

	CCGGTATGATACCCTCCAG<br>TCAGCCGCATTACCTTTAG<br>GACTCTCATAGGACCCGAT<br>AAGTTCTCTCCTATAAGAC<br>CTCCT	
EmauSat6-241	GTTTCATCCAATTTGGAGT<br>AAATACCCTAACTTTGACC<br>TATTTAGGTCAAATAGGCC<br>ACACTTAGGGTTTTGGTCTT<br>AACTAAGCTCAAAGTTTGA<br>CCAAACCCAATGGGTCCCA<br>AACTTGTCTAGTAGGCTTCT<br>AGTTATGTGTGAAAGTTTG<br>TGGGAAGTTTGGTCAAATT<br>TTGAGATATTTGACCAAAC<br>CCTAAGTTTGGCCTATTTGA<br>CYTAAATAGGTCAAAGTTA<br>GGGTTTTTAG	241
EmauSat9-289	AAACAACTCCAAATTAGAC<br>CAAACTTCCCACAAAATTT<br>CATACAAATACTCGAAGCC<br>TACAAGACAAGTTTGGGAC<br>CCRTTRGGTCTGGTCACACT<br>TTGAGCCTAATCAAGACCC<br>AAACCTGTGTAGTAGACTT<br>GAAGTAAATATGTGAAAGT<br>NNTAGGATGTTTGGTCAAA<br>TTTCGAGAAATTTGACCAA<br>ACACCCAAACTTTGACMTA<br>TTTAAGTCAATTAGGCCCC<br>ACTTAAGGTTTGGGTCTTG<br>ACTAGGCTCAAAGTGTGAC<br>AAGACCCAWTRGGDCCHY<br>AACT	289
EmauSat12-242	AATTKRSTTHTAGTGTATGT<br>GTGAAGGTTTGTGGGAAGT<br>TTGGTCCAATTTGGAGATG<br>TTTGACCAAACACCATAAG<br>CTTATAGACAAGTTTGGGA<br>CCCATTGGGTCCASTCAGA<br>CTTTGAGCCTAGTCAAGAC<br>CCAAACCCTAAGTGGGGCC<br>NACTTGACCTAAATAGGTC<br>AAAGTTAGGGTGTTTGGTC<br>AAACATCTCCAAATTTGAC<br>CAAACTTCCCACAAACCTT<br>CACACATACACTA	242
EmauSat42-247	TTCTAGTATATRKGTRAAA<br>GTTTGTGGGAAGTTTGGTC<br>CAATTTGGAGATATTTTMA<br>CACATACCCTAAAAGCCTA<br>TTAGAYAAGTTTGGGACCC	247

	ATTGGGTTTNGTCACCCTTT GAGCCTAGTCAAGAGTAAA ACCCTAAGTGGGGCCCAAT TGACCTAAATAGGTCAAAG TTAGGGTGTTTAGTCAAAC ATCTCCAAATTGGACCAAA CTTCCCACAAACTTTCACA CAKACCTATTTAGGTTAA	
EnocSat432-481	GTTCTTGGTAAAGCATAGC AAGCATAGAATTCCGTCGT CTGTAATGAGGGGCGGATG GATTGACGAAGCAGAGTCG TCTCCATTGAGAGCCGGAT GAAGTGAAGATGCAGAAG AAGAAGCCATCTTAGAGAG CAATGGGATATGGAAAAGG AATTGGATATGGAAGAGGA TAAACGAACCGGTATGTTT ATAGGAGGAGCTAGGCCAT GGAATTAGGTTCATACTCC AGAATAGGAACGCGTGCCT GCTGAATCTCTAGATCACG TGTACTATTTAAATGGGTTT TAAAGAGTATTTCATACGA TTTTGAGCTGGCAAAACCG AAATTAACAAAATGTCATT TAGATCCGGTGGACCGGAT TTGCACCCCCCCTGCGCAG GCGATACGCGTGCACGCAC ACCCGATTAATAAGTTTCC GGACCGAATCCGGTTGGCT AAGTGCGAAGTCACTGCGT TTATATTGTCGAACCTCTTT TTTTT	481
EnocSat283-134	TGTGAACCCGCATGGTATT CTCACTCCTTTCCGCTCCTT TTTCTACAGGATGTGAATC CGCATGGTTTTCCTGTTTCC CTGGTTAGGACGTGAGTCC GCATGGTTGCCCTAACCAT GAGCCGTTAGGTTTGAGA	134
EnocSat459-131	CGTGTTGACTTACATCCTA GCTCGCGCAGCCCACAATC AGGAAAGCGTGTTGACTTA CATCCTGTCGCAGGGGAAT AGGAAGGCGTGGAGACTTA CATCCTATTCCTAAAAGGT AAAAGAGACTAGGAAAG	131
EnotSat4-233	GGGTTTTACATACGTACCA TTCTAAACTTTGGAATTCAC CTATTTCAAACCTAAACTCT CATATTTACATATCTCACAT	233

	CAAACATAGCACTACATCA<br>TTTTTTCTTCAAATTAAAGA<br>AACACAACTTTTAACCATA<br>TGAAAAAGTGTGAAACATG<br>TAAATATCAAACTTTGGGT<br>GTGAGATAGGAAAAATCAT<br>AAGTTGTAGGTGCTATGTA<br>TGTAAAAACTCCAATTAAA<br>G	
EnotSat89-100	CCTATGAGATTCCTGAAGA<br>AGATCAAAGTGACTAATGA<br>GGAGAGAACTTATCGGGTC<br>CTCATAGCCAGCAGGAGAT<br>CTTATAGGAGAGAACTTAT<br>CGGGT	100
EnotSat130-119	TAGGGATTCAGAGGCTAGG<br>GTGTGTTCTAGCTTGAATC<br>ACACTACAATGTCTTACGG<br>GTATGTGTTCCCGCCTCGA<br>CAAAGGATCAAATGGGTTT<br>GTGCTCCCATTGATCAAAG<br>AGTAG	119
EnotSat152-105	TTCACCACCGCCTCCTCCTC<br>ACATTTACAACTCCCCACC<br>TCCTCCTCCTCCTTCTCCAT<br>CGCCTCCTCCTCCCTATATT<br>TCCAACCCACCACCTCCAC<br>CTTCTCC	105
EnotSat1-231	CAACTCCCTTKMKDCBGGA<br>GTTGAKCCDCWTGTSTTTT<br>CCWWCATTGTGAVNTGAA<br>GAAAAAAAGRAAGAVGAG<br>ATGAAMGAGAAAGACGAA<br>GGAGCAGAAGAAGAAGMA<br>GAAAAAGAGATCGGAAGA<br>GCACACGTCTGAACTCCAG<br>TCACTCTCGCGCATCTCGTA<br>TGCCGTCTTCTGCTTGAAA<br>AWAAHAKWGRAGKAATGC<br>YTBGSTGGSKSKBGGHTCTT<br>TTTCTGT	231
EnotSat2-224	CMTMCAARAWCTADATAC<br>TAACCTAATCCMWYACCTC<br>RCTCTTTCCWHTTCGKTTTC<br>TCTYCTCCCTCTTCTTCTCC<br>TTCTCTCTCTTCCTCCTCTT<br>CTCCTTCCTTCAGATCGGA<br>AGAGCGTCGTGTAGGGAAA<br>GAGTGTAGGATAGGGTGTA<br>GATCTCGGTGGTCGCCGTA<br>TCATTAAAAAAAAAAAMA	224

	TTNYATAACCCACANCCAH MTTTTGCTATGATA	
EnorSat15-183	ATCTGAAAATGCATAAACA GGCAGCCACAGTAAATTAA GCATAACTCCTTGCACAAG TTACGGAACTGAATTATTTT TGATGTTTTGAAAAGCTTA CGGAATCCCCTACAACTTC TATGTTGATCATTTCTCCTA ATAAATGCTGGTTTGATAC CAGTTTTAAGAAAAGCTTA AATTTATTTA	183
EnorSat43-1114	CGGCGCCTAGGCGCCCCCT CACATAAAGCGGCGCCTAG GCGCCAAATCTCCGGCGCC TAGGCGCCATATCCAGTCA GCGCCTCGCCTGCCGCGCC TGCTGCCGCGCCGCCTGCC GCCTGACGCCTCGCCTGCC GCGCCGTCAGCACCGCCAG CCGTGCCCCATCTCCGCCG TCCACGTGGCGTCCATCCG CGCCGCCACGTCAGCGATC CACAACTCCTCCCATCCTTT CACCTGGTGCTGCCACGTG GCATGCCACCTCACCCGGA CCGCCACCTCAGCCGCCAC GTGGCGCCCATCCAGGCCC CCACTGCCACGTGTCGCCC ATCCAACGGCCCCCCTACC TATAAATACCCCCCCATCT CCCTCCTTTTAACCCTAATC ACCCTCATACAACCTCCCC CCAACTTCATCCCCTCCAG CTCCGGCGAGCTCCCCACC GGCGCCACCTCCGGCCACC GCCTCCGGCCACCGGCGAC CGCCTCCGGCGAACTTTTC CGGCGACTTTCCGGCGAGT TTTTCCGGCCGCCTCCGGC GACCTCCGGCGAACTTTTT CCGGCGAACTTCCGGAAGG CCATTTTCGGCCCCCTTTCC AAGCGTCAAACTCCGGCGA CCTCCGGCGAAGCTCCGGC GAACTTTCCGGTGGGTCTC GCCGCCTCCGGCGAACTCT TCCTAAACTCAAAAAACTC CTTCTTCCCCCTAAGTACCC GCGTTGGTTCGTCCGCCGG GGAAATGCTATGTCTTTTCC AGGTACCGCCAAGGAGAAT	1114

	AAAGACCCCCGTGTTGGTT<br>TGTCCGCACGGGTGGATAT<br>TCAACAAAAAAATTAGACC<br>CTAAAGCCTCCTTGGGGAG<br>GTGACGCCGGCAGGCTGCC<br>GAAATTTTGACCCGAATAT<br>GGCCTCGGAACGGCTCGCG<br>GGTGGCCTTTGAACGGCCC<br>TGGACTTAAACTAAAAAAG<br>CCAAAAAAATTTAAACCCC<br>TATAAAAGGGCTTAAAAAG<br>GCCAAGTCTTCGGCCAAAA<br>ATTCAAAAAAATGAGAAAA<br>GCGCCAAAAACTGGGGTCG<br>GCGCCTAGGCGCCGAACAT<br>CTGGCGCCTAGGCGCCCCA<br>CTCTCGGTTTCAAACCCGA<br>GAGGTGGGCGCCGAGGCGC<br>CGAAATC	
EnorSat66-103	GAAAGAGGATGTCCACTAA<br>GGGCTTATCCTGTTTCAAAT<br>AGTCAAGGGGAGATCTTCA<br>TAAGAGGGATTGGGACCGA<br>TCCTATAAGAGTCCTAAAA<br>GGTTAAC	103
EnorSat82-126	TCGGCCCGAAGGGGAGGAT<br>AGGAAAGCACGGGGACTC<br>ACATCCTATCCGGAAATGA<br>GACTAGGAAAGCGCGGGG<br>ACTCACATCCTAGCTCAGG<br>CGAGGGTCACGAGAAAGC<br>ACTGGGACTCACATC	126
EnorSat167-144	TACTACAAATCACCTCCCC<br>CACCATCACCATCACCTCC<br>TCCACCATACCATTACCAA<br>TCCCCACCCCCACCATCAC<br>CTTCTCCTCCACCTCCCTAT<br>CACTATAAGTCTCCTCCTCC<br>TCCATCTCCTTCACCACCTC<br>CTCCATAC	144
EnorSat47-386	TTAWAAWWWATARYYWC<br>AMRWGTTBADVVGAGHGA<br>YAWAMABARBVMTWTVTK<br>AAATTAAGGGTTCGTTTGG<br>CCCAGCTGTGCTNAGAAGA<br>NTCTNNTGTCAGAAGAATC<br>TGCTGTCAAATTTTGTTCTT<br>GTTTACTGTTTGGTGTCTGT<br>TTGGAAAACTCAGCTGTGT<br>GACTTTGTCWGCTGTCAGA<br>AGAATCTGCTGTCAGAASA<br>GCGGGGCCAAACGAAGCCT	386

	AAGTTTAHTTTGGAAGATH<br>TGRWTTWWGTYYWWKTR<br>MWTWWMYWSWMKKCAG<br>TGCTCAAGGAAGCAATTGA<br>TGATCTGGAATGGCCCGGT<br>TCCAGCGTCCAGATACGCA<br>TACAGCCTGATCCTCCCTC<br>AGTGACTTTCAGAGCTGAG<br>GGCCATGGCGACT	
EnorSat1-314	GAATTCTCTCTYKMWYTCY<br>CTCYCTCTHTCYCYCTATCT<br>CTMTTCTCTCTCTCTCTCTC<br>TCTCTCTCTCTCTCTCTCTC<br>TCTCTCTCTCTCTCTCTCTC<br>TCCCTCTCTCTCCCTAAAGG<br>AGAGAGAGAGAGAGAGAG<br>AGAGAGAGAGAGAGAATT<br>CCTACCTAGGTAGAAAGAG<br>AGAATTCATCTCTCTCTCTC<br>TCTCTCTCTCTCTCTCTCTC<br>TCTCTCTCTCTCTCTCTCTC<br>TCTCTCTCTYTCTCTCTCTC<br>TCTCTCTCTCTYTCMCTCTC<br>TCTCHCTTKYYTKWMTTCT<br>CTCYCTCCCTCTCTCTCTAC<br>C	314
EoctSat16-170	CAATTTGACCTAAATAGGT<br>CAAAGTTAAGGTGTTTGGT<br>CAAACATCTCCAAATTGGA<br>CCAAACTTCTCACAAACCT<br>TCACACATACACTAGAAGC<br>CTACTAGACAAGTTTGGGA<br>CCCATTGGGTCCGGTCACA<br>CTTTAAGCTTAGTCAAGAC<br>CCAAACCCTAAGTGGGGC	170
EoctSat49-100	CTCTCCTATAAGATCTCCTG<br>CTGACTATGAGGACCCGAT<br>AAGTTCTCTCCTCATTAGTC<br>ATTTTGATCTCCTTCAGGAA<br>TCTCATAGGACCCGATAAG<br>TT	100
EoctSat42-129	TTTGATGATCCTGGGCACG<br>CGGATCTAGGAACCCCTTT<br>TGGTATCAAGTCCTGGGCA<br>CGCGGACTTGATCACCTTA<br>CCTTTCAGGGCCCCTTCATA<br>CCCAGCTATGAAGGATAAA<br>AACCACCCTAATTT	129
EoctSat108-171	TCAGTGGCACCCTCTCATT<br>AGTCATATTTCTTCTTGACC<br>CAAGGTTGGAGCAAGCCTT<br>AAGTTGGCACCTTCCAACC	171

	AGTCACTCCTTTGGATTCCT<br>CTAGGATTGGAGTAAAGCC<br>TTGCAGGGGCACCTCTCCT<br>ACGAATCCATGGCTATTTG<br>AGCCTGGGAGCAAGCCC	
EoctSat395-120	CAGGTTGGTCTTTCTGTCCC<br>GCTACGGATCGACCCCTTG<br>TCTGGTTGTTCGAAGATTA<br>GGCTGACTTCTACCGGTCA<br>CCACCTATTCCTAGATCAG<br>GCATTGGACACCTTTTACT<br>ATGCA	120
EoctSat434-387	ATCACCAACCTGATCTTTC<br>ATCTTGCCATTGCCACCAA<br>AATCACCCTCATCCTCTTCC<br>TCATTCTTCTTCTTCTTGTA<br>GCTGCCTCCCCATTCCTTAT<br>TCCCACGACCGCCGTCTCT<br>CTCCTCCTTGTGGAACTCAT<br>GACCTCTCACTTTTTTCTTC<br>TCATCACGACCACTGCTTC<br>GCTTCTCCTCCTCGTTTTTC<br>TTGCGATTGCCACCACCCT<br>CCTCCTTCCCGTGGCCACTA<br>CCTCCCTCCTCTTCCAACCA<br>AAAACCACCGTTCCAGTAC<br>TCCATCTCCTTCTTCTGCTT<br>CTTCTCGTCATCAGCTCCCT<br>CCTTCTGCCAGCGTACTCC<br>ATCCATTATATTCTTTACAC<br>CACGACTACACTCTCCCCT<br>CTCTTTCTTCTCGCC	387
EoctSat57-510	CCTAACTTAAACTTATCCA<br>AACTCAACCAAACTTTGCA<br>CAACCACYYWYMMCATCA<br>TAGACVACCCATCMATGGT<br>CAATGGTTGMCAWGGTCA<br>ACCCTGKTYRACCCAGYYC<br>CTTCACAAACCCTAWTTTT<br>GACCTAAACCCTAATTAAG<br>GTCAAAACCTAGCCTAAAC<br>ACATCCACATGCCACCAAA<br>CTTGGCATGGGGTGTTTTC<br>ACATCATTGRCGACCCACC<br>CATGGTCAAAGGTTGACCA<br>GGTCAACCCTGGTCGACCC<br>AGTTCCTTCACAAACCCWA<br>TTTTGACCTATTTGGGTCAA<br>AATKAGCCTATAAGCCTAC<br>CCAGGCTAATTAGGCCTGC<br>CYCATTGCATCCATTAGCA<br>CCCAAAGAGACCCCTRGRC	510

	TTACCATGGGAAGCCTGGCCAACCSWCWWCCTTCACAAACCCWATTTGACCACTATGGGTMWMMTWKGGTTTTAAAACCTACCTAAGCTATTTAAACCTATCCAATTGCTATCACTAACACCTAAACACCC	
EparSat66-118	TGACCTATGGGAACACAAACCCATTTGGTCCTTTGTCAAAACGGGAACACATACCCGTAAGACATTGTAGCGTGATTCAAGCTAGAACACACACTAGCCTCTGAATCCCTACTATCTT	118
EproSat103-100	ATGAGATTCCTGAAGAAGATAACAATGACTAATGAGGAGAGAACTTATCGGGTCCTCATAGTCAGCAGGAGATCTTATAGGAGAGATCTTATCGGGTCCT	100
EproSat88-222	CTGAGGGCTTGCTCCCAAGCTCAAATAGCCATGGATTCATAGGAGAGGTGCCGATGCAAGGCTTGCTCCAATCCTAAAGGAATCCAAAGGAGTGACTGGTTAGAAGGTGCCGGTGCAAGGCTTGCTCCAACCTAACCAGTACTCCAACCAACAAGGAAGGTGCCGACTGGGGGCTTGCTCCAACCTTGGGTCAAAAGAGATATGACTAATGAGAGGGTGCCA	222
EproSat110-193	ATCCTCTCCTCATTAGTCACCTTTTGACCCAAGGTTAGTGTAGACCCTTAGTGGACACTTTTCCTTGACTGGTCTTAGCCCTGTTTGGATCAGTACGATACCCTCCAACCAGTCGCATCTCTTTTAGGACTCTCATAGGATCAGAGTACGTCCCTCCTATAAGATCTCCCCCTGACTATTAGGATCAGAACAC	193
EproSat35-376	ACAGCTACTTTTTCCATCACTCACACAGCTGAGTTTTCCAAACAGAAACAAGAACAATTTYRKMSHBHGAATYAGCYGACCTCWAAAGCCGGGCCAAACGGAGACTTAGGCTCWGTTTGGCCCAGCTTCTTCTGTCAGCTGTCAGCAACTTCTTCTGACAGCAACTTTTTCCATCACTCACACAGCTGTC	376

	ACACAGCTGAGTTTTCCAA<br>ACAGAAACAAGAACAATTT<br>CAGACAGCAGCTTTTCCTG<br>TCAGTNCAGCACAGCCGGG<br>CCAAACTGACCCTAAGTCT<br>AMGTTTTGCCCAAATTTTT<br>ASNGAATCWGTCAGCACTT<br>TBTTCTGACAGCDACTTTCT<br>CCATCACTCACACAGCTGA<br>GTTTTCCAAGCATA	
EproSat70-931	TATAATGTCCCGTATCTGAT<br>GTGTAGGCTSTRYMRRKRS<br>AWKKAWYMWRYWAWMK<br>MAAWTATWTKWKGTYKW<br>AKTMAAAMAWWTWARGK<br>TWAWTWAAAWTKRAMRS<br>WRRRRYMWWAWAARYWA<br>ACTTACGTGTCTCCCTCAG<br>GCACAATDGTBATCCTCCC<br>AGCAGGAACTCGTGCTGGT<br>GCCTCTGGCTCTGGMKGCG<br>CAGGAGGTGTNNNCAGGCG<br>GGCATGCATCAGTMGTGGG<br>TTGGGAAGGAGKGAGCTGG<br>RGGAGTGGTACAGGCTGTA<br>TGGGTGGGGGCTGTGAGGG<br>TGSTATGGGTGGGACCTGG<br>GAGGGTGTWAMATAAGTA<br>GGGGGTCGAGGTGGGGGCT<br>GAGGAAGTGGTGCGTACGC<br>TGTATGGTATGTGGGTGGG<br>GGGTAGGRAGGTGGGTAGT<br>ATGGATATAGGGCCRGGTC<br>GTATGNGNGGNNTGCAAAT<br>TACAGGTACGCGTCTCACC<br>ACCSCTACTGCCACTACTGC<br>TCCTACTACCTCACCCACC<br>ACCACCACACAGACCACCC<br>CTACCGCCTCTACTTCTCCT<br>ACTACCTCGCTCACCACCA<br>CCACACCCACCACTCCTAC<br>TGCCTCTACTTCTCYTACTG<br>CCCCCCCCCCCWCCACCAC<br>AACACAYACCCGACWACC<br>CGATCCACCCTCAGCCCCA<br>CTTCCYACCCCTTCTGACGC<br>SCCACYTAGTTGGCCACMC<br>TACNCACCTACATACGACC<br>CAGCCACATATCCTTACTA<br>CCCCCATCCGTACCCCCCA<br>CACCCATACCATMCTAGYT<br>ACSCWYYWMTTTCYCAGT	931

	CTTCACCTCRGMCKMCCCC<br>TTTYTTKCCASCGRYYCGR<br>GTCCCACCCATACGACCCT<br>CCCATCCCTATCCCACACC<br>ACCCTCCCAGCCCCGACCC<br>ACACCACCCTCCCACCCAC<br>ACCCTATAGAGCCGGTACC<br>CCAGTTCCA	
EramSat12-128	CCTAATCCCTCTCTCCACTT<br>AGCCAAAATCGGCCAAGGA<br>CCCTTGCTCCCCTCTCATCT<br>CCCTTAGCCGACCAACCCT<br>CTTCAAGCTTTTAGCCAAC<br>CCTAGCTCCTCTTCATCTCC<br>ATAAACCAAAC	128
EramSat15-105	TTCTCCTTCAGCAACAATCT<br>CTTCATCATCAAATTCTTCT<br>TCTTCTCCTTCTTCTTCTTCC<br>TCTTCTTCTTCATCTTCGGA<br>GCCAAGTTCTTCTCTTTCTT<br>CATT	105
EramSat74-170	ACTTTAAGATTAGTCAAGA<br>CCCAAACCCTAAGTGGGGC<br>CAATTTGACCTAAATAGTT<br>CAAAGTTAAGGTGTTTGGT<br>CTAACATCTCCAAATTGGA<br>CCAAACTTCTCACAAACCT<br>TCACACATATACTAGAAGC<br>CTACCAGACAAGTTTGGGA<br>CCCATTGGGTCCGGTCAC	170
EramSat1-266	ATTAGTSRMKAKTWCTGAY<br>ATTAGATCTGTGGCTTTCTC<br>GWCTGKGGTGGGAGGGAG<br>AGAGAGAGAGAGAGAGAG<br>AGAGAGAGAGAGAGAGAG<br>AGAGAGAGAGAGAGAGAG<br>AGAGAGAGAGAGGGAGAG<br>AAGATCGGAAGAGCACAC<br>GTCTGAACTCCAGTCACTA<br>ATGCGCATCTCGTATGCCG<br>TCTTCTGCTTGAAAAAAAA<br>AAAGGGGGGGGGAGGGGG<br>GGGGGAGGGGGWSMGMW<br>MMMKCACCGTCGGCAACC<br>AGGAGATCG	266
EramSat2-233	TACGGCGWCSAGTKCKAT<br>MCTVDCTAYCTTCCTTCCY<br>CYCTCTCTCCCCCCCTCTCT<br>CTCTCTCTCTCTCTCTCTCT<br>CTCTCTCTCTCTCTCTCTCT<br>CTCTCTCTCTCTCTCTCTCC<br>CTCCCTCTCTCCCAGATCGG	233

	AAGAGCGTCGTGTAGGGAA<br>AGAGTGTAGGCTATAGTGT<br>AGATCTCGGTGGTCGCCGT<br>ATCATTAAAAAAAATAAM<br>GCASAGTAAMRAARCRGYG<br>AV	
EramSat6-305	AGGGATTCTGGGGGACGGC<br>CGMWWKRWWRMRGTSSA<br>YMARYWSKTHHTCVTHDA<br>WAAACTCCANGAGAGNGA<br>GTTTNGNTTCTTWGRGWKA<br>SAAAAACTYBTACBCGAAA<br>ABAGAGAWWTACGCWGA<br>ARTWATAGAAGSAGAAGA<br>ACCATCACCGYCAAAAGGT<br>AATTTTCCTCTTCTTCTTCT<br>TCTTCTTCTTCTTCTTCTTCT<br>TCTTCTTCTTCTTCTTCTTCT<br>TCTTCTTAGATCGGAAGAG<br>CACACGTCTGAACTCCAGT<br>CACTAATGCGCATCTCGTA<br>TGCCGTCTTCTGCTTSRAAA<br>DR	305
EramSat7-345	CTCCCCTCTCTCTTCGGCCT<br>GCTGTASYYSMKYSCKMYB<br>CCCTASAARTTTYCCWHCA<br>CTAGTTCTTNCNTTTCTTTT<br>CTCTTTTTCTTCTCTTTTTTT<br>TCTACACCACCTCCAGAAG<br>AAGAAGAAGAAGAAGAAG<br>AAGAAGAAGAAGAAGAAG<br>AAGAAGAAGAAGAAGAAG<br>AAGAAGATCGGAAGAGCG<br>TCGTGTAGGGAAAGAGTGT<br>AGGCTATAGTGTAGATCTC<br>GGTGGTCGCCCTACCBBAC<br>CHAYCYWYCTYYYYYYYY<br>YYYTYYSMYTCCSMCKYCY<br>TYYTTTACTTCCCCCTCCAT<br>TTTTTCTTCTTTCTTCCTCTT<br>TTTTTTTCTTTTTTCTTTTT	345
ErepSat5-183	CCGCGTACAATTTCCAGAA<br>CCACCCATCTACTCGGAGT<br>ATGCTTCAGAAGGAGAAGA<br>AGAAGAAGAAGAAGAGGA<br>ATATGAAGACTGACGAGAA<br>TAAAAGAACTTATTCTTGT<br>CATTATGTATTTTGTTATTT<br>CTGTTTTTATTTTCGCAAGA<br>ACTAGTTAAATTTCCATATC<br>TTGTATCTGG	183

ErepSat4-192	TTGACTTGGAGACAAATTG<br>AAGAAGCTTCAGGAGAAGT<br>TGATCGTAGATGGACAAAA<br>ACATTTAGCAGAAAGAGGA<br>GTACATCCATTACGAGACC<br>TCCTGTTATTGAAGAAAGC<br>GAAGAAGAACTTGGCTCCG<br>AAGATGAAGAAGAAGAGG<br>AAGAAGAAGAAGGAGAAG<br>AAGAAGAATTTGTTGATGA<br>AGAC	192
ErepSat6-170	GACCCAATGTGTCCCAAAT<br>TTGTCTAGTAGGCTTCTAGT<br>ATATATGTGAAGGTTTGTG<br>AGAAGTTTGGTCCAATTTG<br>GAGATGTTTGACCAAACAC<br>CTAAACTTTGACCTATTTAG<br>GTCAAATTGGCCCCACTTA<br>GGGTTTGGGTCTTCACTAA<br>GATTAAAGTGTGACCG	170
ErepSat48-100	GAGATTCCTGAAGGAGATC<br>AAAATGACTAATGAGGAGA<br>GAACTTATCGGGTCCTCAT<br>AGTCAGCAGGAGATCTTAT<br>AGGAGAGAACTTATCGGGT<br>CCTAT	100
ErepSat121-469	ACGCCATCCGAGGTGCCAA<br>TGCAGCGTCACGCCGGATC<br>CGTGGGGCCCGTTTGGCTG<br>CGGCACAAAAATCGGCGTA<br>ACTCTGCGTGCAGGACTCC<br>GTTTCGCTATTATGACCCCA<br>TTTCGGATACGCCGGGGAG<br>AGCCCGTAGACGTGACGAG<br>TAGCCGGAGGCCACCTCGA<br>CCACGCCCGCGGCGCACGA<br>TTCGGAAGTGATGCGGCCC<br>CGCAGCACGGAGAAGTTTC<br>GTACAAGTATATTATATGC<br>TCATATTCATGCGGGTGCG<br>ACCATACCAGCACTAGTGC<br>ACCGGATCCCATCAGAACT<br>CCGCAGTTAAGTGTGCTTG<br>GGCGAGAGTAGTACTGGGA<br>TGGGTGACCTCCTGGGAAG<br>TCCTCGTGTTGCACTCCTTT<br>TTTGCCGCAGATAGTTTCGT<br>GCAGTCTTTTCCCGCGCCC<br>GTCAAAATTTACCACCCAC<br>ATCGCGTCACGGAGGTCAT<br>CCACGGAGGG	469

ErepSat269-232	TCCTCCCTCCTCTTCCCTCA<br>CGTGGAACCAGCACAACCA<br>CCCCCTTCTCCTCCTCTTCA<br>AAAACCGAGCCCAGCACAG<br>CCCCCTTGGGGCTCCTCTTC<br>ACAACTCTCTCCATTTTCCC<br>TCTCCTTCCTCCTACAGCTC<br>TCCCTCTCACGCCACCTCCT<br>CCTCCCCTTCAAAAACCGA<br>AACCAAAGCAGCCCCTTAC<br>CTCCTTCCTCCTGCAGCCAA<br>AACCGAACCAGCCACC	232
ErepSat283-417	AAGGCAACTACAAGAAGA<br>AGAAGAATGAGGAAGAGG<br>ATGAGGGTGGTTTTGGTGG<br>CAATGGCAAGAAGAAAGA<br>GGTTGGTGATAGCGAGAAG<br>AAGGAGAAAGAAGATGTT<br>AGCGAGAAGAAAGAGAGG<br>GGAGAGTGTAGTCGTGGTC<br>GGTGGAAAGAATATACAAA<br>GGAGGGAGCTGATGACGA<br>GAGGAAGCAGAAGAAGGA<br>GATGGAGTGGAACAGTGGT<br>TTTTGGTTGGAAGAGGAGG<br>GAGGTAGTGGCCGCGGGAA<br>GGAGGAGGGTGGTGGCAAT<br>CGCAAGAAAAAAGAGGAG<br>GTAGGCGGTGGCGGTGGAG<br>AGAAGGAAGAGAAGAGAA<br>GCAATGGTCGTGACGAGAA<br>GAAAAAAGTGAGAGGTGA<br>GTTCCACGAGGAGGAGAGA<br>GACGGCGGTCGTGGGAATA<br>AGGAATGGG	417
ErepSat1-330	AATTTGTTTCATGTATTTTT<br>CATTCTYYWWYWWMADC<br>GMTCAAWTDTGTATGCTW<br>ACCTGCATTTCATMGRMTC<br>TGWAVAAWCAAWATTTGA<br>AGAAGAAGACWGAGAAAG<br>AGAAGAAGGAGAAGATTA<br>GTCTTCTGTTTTTTCCCTCT<br>CTCCCCATTCTTCTCTCTCT<br>TCTTCTCCCTCTTTCTCTTCT<br>TCTTCTTCTTCCTCTCTTTCT<br>TAGATCGGAAGAGCGTCGT<br>GTAGGGAAAGAGTGTTAAG<br>ATTAGTGTAGATCTCGGTG<br>GTCGCCGTATCATTAAAAA<br>ARAAAADRWTTTAADSTA	330

	WMMMTASSMTHKWKTGTC<br>TACGCTCA	
ErepSat2-304	YWKRMSWRKRDGHDCATG<br>ARWTCTCVGTSTWKTRMTK<br>STCTGSGBKKRBVAAASMR<br>GAAGACGGCATACGAGATR<br>GCTTCAGGTGACTGGAGTT<br>CAGACGTGTGCTCTTCCGA<br>TCTTTCTTTTTCTTCTTCTCC<br>TTCTTCTTCTCTTTCTTTCTC<br>TTCTTCTCCTTCTTCTTCTCT<br>TCCKATCTGAGATCGGAAG<br>AGCACACGTCTGAACTCCA<br>GTCACCTGAAGCTATCTCG<br>TATGCCGTCTTCTGCTTGAA<br>AAAAAAAGGGAGKGGGGG<br>ATSGGRRGGGRGGGGGMA<br>CCTCCGAAATGAGAT	304
ErepSat3-357	AGTTCAGACGTGTGCTCCG<br>CGCGYCTCCCGCGASWGRC<br>CNGCCCGCCTGCSCCCCCG<br>GGCGCCATTTTTTGCCVAA<br>AATTCCGATTTTGCCCCCAT<br>TTTTTVGCCAATTTACGCCT<br>CCTGACCCCTCTCCTTCCTA<br>TAAATAGCCCCCTAGGGKC<br>TAGGGGAAGGTTGCACCCT<br>CCCCTCTTTCTCTTCTTTTCT<br>TCTCCTCCTCTCCTTCTTGA<br>GACTCGGGTCTTGGGATCG<br>GAGGAGCACCTCSTCTGAA<br>CACAGGGTGTTTAGATCAG<br>TGTAGGGCGGGKCCYAAGA<br>TGYCAAGAATWCTKSAAAA<br>TCAGGGGBGTCADGDTBDD<br>CATVHTVKSAAAAMAATGG<br>AAACTGGAC	357
ErepSat24-298	CTAGAAAGGTGATATTTCC<br>TCCTGAGCAGTGACAAATT<br>GTCACTGGTCAAAAAAGAS<br>GCSCCYWGCGACAGCTACC<br>CCTCCTCTCCTCWTCYCTTA<br>TTCTTCCTCTTCNNTCCAGA<br>CTTCGGATCACCCATTWAT<br>GCGGGAAAAACACTCCTGA<br>AGGTCAAAATTCAAGTCAG<br>AGACTTGATTTTCAGGAGA<br>GGGGAAGAAGAGGGGAAG<br>AAGAGAGGAAGAAGAGGG<br>GAAGAAGAGAAGAAGAAG<br>AGRAGAAGAGGAGAAGAG	298

	GAGTAGAAGTGGAGTGATT<br>HHTMSSMAGGAAGGG	
ErepSat9-398	CTTTCCCTACAYSMMRMTC<br>YWYMGGWYRTHHTHTGTH<br>DBGDDATACCGDCHGCHHT<br>TCHDADTWNNCCTCGAMC<br>CWTACCWKKGAAKTTCHC<br>AAAWATTTWTDCAAWHAA<br>YATTTCGCARTTGVWYSCC<br>AAAAACTCKTGAGAAGGCC<br>CCATTTAATWCCCAAAAAC<br>TCTTCAAATHCCCCCCCTAT<br>GCCTATAAATAGACCCCCA<br>AAGCCTCAAAGGGGGGCAC<br>ACCTTCCCCCTCTTCCGAGG<br>TCCTCGTTTGCATCCCCTTC<br>CAGACTCCRGTATTCCTCTG<br>CATAGGGGTCGTATTTGAA<br>GAGTTTTTAAGGAGTAMTT<br>GGGGRAAWYGMAGRGTYT<br>TAGRTTCCRGACDVVHTTH<br>BGHBYKWMMTYYWKTGG<br>CATCCTCGCTCGCAGCTTCC<br>C	398
ErigSat36-168	CTCGTCTTGGCCTGGACGA<br>TCCTCCTCGTCTTCGTCCTC<br>GTCTTCAGAAGATTCATCT<br>CGAACCTCGTTGACGATGC<br>CCTCCCCGTCTTCGTCCTCC<br>TCTTCGAAGGCGACTGCGA<br>CACCCTGGCGTTCATCGAT<br>CTCTTGGTCATCGTCAGCA<br>CCGTCCATCTCTTC	168
ErigSat52-170	TTAGGTCAAATTGGCCCCA<br>CTTAGGGTTTGGGTCTTGA<br>CTAAGCTTAAAGTTTGACC<br>GGACCCAATCGGTCCCAAA<br>CTTGTCTAGTAGGCTTCTAG<br>TGTATGTTTGAAGGTTTGTG<br>AGAAGTTTGGTCCAATTTG<br>GAGATGTTTGACCAAACAC<br>CTTAACTTTGACCTAT	170
ErigSat1-216	ATCTACACCSAGATCKACA<br>CWCTTTCCTTSCGCCCCGCC<br>ATGCMCAACACGAAGMAG<br>AAGGGGAARAAGAAGAAG<br>AAGAAGAAGAAGAAGAGG<br>AAGAGGACGAGGAGGAGG<br>AGAGATCGGAAGAGCGTCG<br>TGTAGGGAAAGAGTGTAGG<br>ATAGGGTGTAGATCTCGGT<br>GGTCGCCGTATCATTAAAA	216

	AAAAAAAADTTGCAVBHTG<br>SGGCASGSNT	
ErigSat2-249	TTTTCAAGCAGAAGACGGC<br>ATACGAGATCGAGTAATGT<br>GACTGGAGTTCWGACGTTC<br>GCTCTTCCGRTCTAARACG<br>AAAAAGAAAAAGAGGAGG<br>AAGAGGAAGACGAGGACG<br>AGGAAGAAGAAGAGGAGG<br>AGGAGGAGGAGGAGGGAG<br>ATCGGAAGAGCACACGTCT<br>GAACTCCAGTCACATTACT<br>CGATCTCGTATGCCGTCTTC<br>TGCTTGAAAAAAAAGTAGA<br>GTGGAGGKSDMGGGGWGK<br>SGGGGA	249
ErivSat1-303	TGACTGGAGTTCAGMMGW<br>SKTBTGCTSTGCTCTTCCTST<br>CTCTCTCTCTCTCTCTCTCT<br>CTCTCTCTCTCTYTCTCTCT<br>CTCTCTCTCTCTCTCTCTCT<br>CTCYCTCTCCCTCCCTCTCA<br>CGAAGGAGAAGAAGAAGA<br>AGAAGGAGGAGGAGAAGA<br>AGAAGAAGAAGAAGAAGA<br>AGAAAGATCGGAAGAGCA<br>CACGTCTGAACTCCAGTCA<br>CTCCGCGAAATCTCGTATG<br>CCGTCTTCTGCTTGAAAAA<br>AGAARGRGAGGGGGTGGG<br>TGGRGGRGCGAGGRGGGM<br>WRSWKTKKRKGMYWGGGT<br>G	303
ErivSat4-195	TCGCGGAGCGMTTTACACC<br>CTATCCTACACTCTTTCCCT<br>CCCCTWCGCTCTTCCGACC<br>TCCGAGAGGGAGGGAGAG<br>GGAGAGGAGGAGGAGGAG<br>ATCGGAAGAGCGTCGTGTA<br>GGGAAAGAGTGTAGGATA<br>GGGTGTAGATCTCGGTGGT<br>CGCCGTATCATTAAAAAAA<br>AAAARABWCRGYASGRMG<br>GCYRGCAB	195
ErivSat5-257	CTAGARRAKHTTAGGHCTA<br>DDGGWATAGMGAAAMTAA<br>ATGKAAWTAAACTTYRAM<br>AACAAATTAATGARCACCA<br>GGCTKARAAGAGGTTGCTC<br>CTTCCTCTTCTTCTTCTTCTT<br>CTTCTTCTTCTTCTTCTTCTT<br>CTTCTCCTCTTCTTCTTAGA	257

	TCGGAAGAGCGTCGTGTAG GGAAAGAGTGTAGGATAG GGTGTAGATCTCGGTGGTC GCCGTATCAKTAATAAGGA AGCAAGDBRTAATRWKCW MAMRWCTAA	
ErivSat2-210	MSRSKYWKSHAATCTAHCC TCTTTHTCTHCWGGGCSCT CTTCTCATCCTATCCCCCCT AAAAACAAAAAAAAAGAA GAAGAAGAAGAGAAGAAG AAGAAGAAGAAGAAGAAG AAGAGAAGAAGAAGAAGA AGAAGAAAGATCGGAAGA GCGTCGTGTAGGGAAAGAG TGTAGGATAGGGTGTAGAT CTCGGTGGTCGCCGTATCA TTAAA	210
ErivSat3-225	GCCTATATAGGGCCCCTTC GCGGCTAAGAGATAATTTC TTCTTCTTCTTCTTCTTCTTC TTCTTCTTCTTCTTCTTCTTC TTCTTCTTCTTCTAGATCGG AAGAGCACACGTCTGAACT CCAGTCACTCCGCGAAATC TCGTATGCCGTCTTCTGCTT GAAARAAAAGGAAGAAGA AGRRRRARGAAGAGAAGA AGAGAAGAAGAAGAAGAA GAAAAGAAGAAGA	225
EsopSat255-250	TCCAGAGAAGGCAACGGG AATACCATGCGGGTTCACA TCCCGGCCTGAAAATCCTG TGGTAGGGCAACCATGCGG ACTCACACCCTAACCAGAG GGACAGGACAACCATGCGG ACTCACATCCTGTCAAAAG AGGGTGACGAGACAACCAT GCGGACTCACGTCTCGACC CTGAAACCCGTGGTTAGGG CAACCATGCGGACTCACGT CCTAACCAGGGGGATAGGA AAACCATGCGGGTTCACAT CCTA	250
EsopSat51-300	GGAAAGCACCTGTGTGAG TGATGGRWARWGYTGCTG HCAGAAGAAGCTRGGCCNA GACTAAGGGTGTGTTTGGC CCMGCTTATCDGCGGCTTC TSCTGTCAGHHACAGTTCA GCTACTTTTTCCATCACTC ACANNNNNNNNNCAGCTG	300

	GGCCATCCAAACGGGCTTT<br>TTCTGAAACAAGAACAATT<br>TCCGTCAGCAGCTTCTCCG<br>AACAGCCACAGTTCAGCTG<br>CCGATCAGCCGSGCCAAAC<br>TGACCCTTAGKCTCNGTTT<br>GGCCCAGCTGTGCTGAMAG<br>AWTAAGBBSCCCTCCT	
EsucSat64-105	AAAAGAGAGACTATTGAAA<br>GAGGATGTCCTCTAAGGGC<br>TTATCCTGTTTCAAATAGTC<br>AAGGGGAGGTCTGATAGGA<br>GGGATATTCTCTGGTCCTAT<br>GAGAGTCC	105
EsucSat174-118	GGTGACCGGTAGAAGTCAG<br>CCTATTCTTCAAACAACTA<br>GACAAGGGGTCGATCCATA<br>ACGCGATGGAAAGACCAAC<br>CTGTAGTGTAGAAGGTGTC<br>CAATGCCTAATCTAGGAAT<br>AGGT	118
EsucSat382-459	CTCTTTTTTCTTGCGATTGC<br>CACCACCCTCCTCCTTCCGA<br>CGGCCACTACCTCCCTCCTC<br>TTCCAACCAAAAACCACCG<br>TTCCACTCCCTTTCCTTCTT<br>CTGCTTCTTCTCGTCATCAG<br>CTCCCTCCTTCTCCCAGCGT<br>ACTCCATCCATTATATTCTT<br>TACACCACGACTACACTCT<br>CCCCTCTTTTTCTTCTCGCT<br>AACATCTTCTCTCTCCTTCT<br>TCTCGCTATCACCAACCTCT<br>TTTTTCTTGCCATTGCTACC<br>AAAATCACCCTCATCCTCTT<br>CATCATTCTTCTCACCACTA<br>CCATCCATCTCATTCGTCTT<br>CTTCTTGTAGCTGCCTCCCC<br>ATTCCTTATTCCAACGACTG<br>TCGTCTCTCTCCTCTTTGTG<br>GAACACACCTCTCACTTTTT<br>TCTTCTCGTCGCGACTACTA<br>CTTCTCTTCTCTTCCTTCTC<br>GCCACTGCCACCATCTACT<br>TC	459
EsucSat390-168	CGGTGGCCTCGGACTTGTA<br>CTTCTCCTTCTCAAGGGATG<br>CGGCAGCTTCTTTCAGTTTC<br>CTTTCTGCTGGTTTTTGCTT<br>TTTTGTATTTTCTTCACCTT<br>CCCCTCCGCTAGTGTCAAC<br>CTTAGTCTTCTTCTCATCAC	168

	CGATGACATCGCCTCCTTT<br>ATTGTTAAGAC	
EsucSat63-303	AACAATTTCARRARKYHDH<br>BTTYWCTGWCAGCACAGC<br>TGAGTCWTAWAAAGACTT<br>AGGCTCTGTTTGGSACAGC<br>TTATTCTGTCAGCAGCTTAC<br>AGCTTCTTCTGTCAGCAGCT<br>TTTTYCATCACTCACACAG<br>CTGTCARACAGCTGAGTTT<br>CCAAANAGNCAAGAACAA<br>TTTCAGACARCAGCTTTTCC<br>TATCAGCAGCTTATTCTGTC<br>AGCACAGCTGGGCCAAACA<br>GAGCCTWAGTCTCWGTTTG<br>GCYTAGMWATGTATKTBC<br>ASTTCTKCDAADTTVTWYA<br>MTKWRGTGATGGAATGAA	303
EsumSat54-119	CAGAGGCTAGGGTGTGTTC<br>TAGCTTGAATCACGCTACA<br>ATGTCTTACGGGTGTGTGTT<br>CCCGTTTTGACAAAGGATC<br>AAATGGGTTTGTGTTCCCA<br>TTGATCAAAGAGTAGTAGG<br>GATT	119
EsumSat187-105	CATTTACTACTTTTCTTTTG<br>GCTGGTATCTCTACCTTCCT<br>ATGCTAGCTATTAATAAAG<br>ACGATCGAGACTGATCCTC<br>TTTAAGCTACGCATAGCAC<br>GCCATTTG	105
EsumSat1-220	ACCCTAAGTGGGGCCAATT<br>TGACCTAAGTWGACTAAGC<br>TTAAAGTGTGACCGGACCC<br>AATGGGTCCCAAACTTGTC<br>TAGTAGGCTTCTAGTGTAT<br>GTGTGAAGGTTTGTGAGAA<br>GTTTGGTCCAATTTGGAGA<br>TGTTTGACCAAACACCTTA<br>ACTTTGACCTACTTAGGTC<br>AAATTGGCCCCACTTAGGG<br>TTTGGGCATTGACTATGCTC<br>AAAGTGTGAC	220
EsumSat2-177	AGAAGCCTACTAGACAAGT<br>TTGGGACCCATCGGGTCYG<br>GTCAAACTTTAAGCTTAGT<br>CAAGACCCAAACCCTAAGT<br>GGGGCCAATTTGACCTAAG<br>TAGGTCAAAGTTAAGGTGT<br>TTGGTCAAACATCTCCAAA<br>TTGGACCAAACTTCTCACA	177

	AACCTTCACACATACCCTC<br>TAGTGT	
EsumSat3-164	AAACATCTCCTAATTTGGA<br>CMTACTTAGGTCAAATTGG<br>CCCCACTTAGGGTTTGGGT<br>CTTGACTAAGCTTAAAGTT<br>TGACCGGACCCRATGGGTC<br>CCAAACTTGTCTAGTARGC<br>TTCTAGTGTATGTGTGAAG<br>RTTTGTGAGAAGTTTGGTC<br>CAATTTGGAGWT	164
EtalSat286-438	GTTGGTTATAGCGAGAATA<br>AGGGTAAAGAAGATGTTAG<br>CAAGAAGGAAGAGAGGGG<br>AGAGTGTAGTCGTGGCCGG<br>TGGAAAGAATATACAAATG<br>AGGGAGCAGGCTGGGAGA<br>AGGAGGGAGCTTATGATGA<br>GAGGAAGCAGAAGAAGGA<br>GATGGAGTGGAATATTGGT<br>TTACGGTTGGAAGAGGAGG<br>GAGGTTGTGGCCGCGGGAA<br>GGAGGAGGGTGGTGGCAAT<br>CGCAAGAAAAAAGAGGAG<br>GTAGGCGGTGGCGGTGGAG<br>AGAAGGAAGAGAAGAGAA<br>GCAATGGTCGTGATGAGAA<br>GAAAAAAGTGAGAGATGC<br>GTTCCACGAGGAGGAGAGA<br>TGCGGAGATCGTAGGAATA<br>AGGAATGGGGAGGCAGCT<br>ACAAGAAGAAGACGAATG<br>AGGAAGAGCATGAGGGTG<br>ATTTTGGTGGCAATGGAAA<br>GGAGAAAGAG	438
EtalSat1-399	AATTGTGTTAMYTWWTTY<br>CYYMAAWTTTTYMARRAA<br>ARKWAAWWARSYWWWW<br>TYMAATAAATACACACAAC<br>CAAGCACTAATCTAAAACA<br>AAAGATTATAAATGAAGTT<br>ACTACTTTTCATTTCATTTT<br>AATAAACTAAAGTACAAGA<br>TAAAAAATAAACATAAATT<br>CATCTAAACTTGGAAATTT<br>TTTCATTACTTAAAAATTAA<br>AAGCATTACAAAAGAAAAT<br>ACAAGATCTAAGCATAAAG<br>AAAGATACAATAAAAATAT<br>AAAGACATTATCACTAATC<br>ATCATATTCTTCTTCTTCTT<br>CCTCTTCTTCTTCATCTTCT	399

	TCTTCTTCNTCTTCTTCTTG<br>TGGAGGAGCATATCTAGTA<br>TAGGTAGGAAGSAAAGAGT<br>GTTAAGATTAGTGTAGATC	
EtalSat27-390	TGTATTGATATGATTTTATT<br>GCAATTTGGAAATGAAACA<br>ACAAAGAACCAACACAAC<br>AAAGAAWTYAAAACCMAA<br>CAAACTTTDTTTTTARNAYT<br>TYAATTTTATTTTTTAATTT<br>TWATGATTTTAGGRTAAAT<br>TATTACAACTCAVACAACA<br>ACGAAATTCAGCTCGTTGG<br>GGGTGGATCTCTGGGTTGT<br>GGATTGAGAARCGGCGGCT<br>GGNTGGAGAGNGGGATAA<br>NGGTTGAATCCACAACCCT<br>AGGGGAGATCCACCCCAAG<br>CTAGCCGAAATCCACAGCT<br>TACCTTCCTTCATCTTYAAA<br>AATCTWMCCTAAAAAAAA<br>MCAAAACCGGAAATAAAA<br>RGRGGAGGBABBTDHVVCA<br>HVAGAAVSMCYMRRRASA<br>GGGAAAACAGGC	390
EtalSat2-559	CAAGAGGAACCAAACCGC<br>ARSRMMMMAVAAAGHVG<br>AHCACAACCGCABCAAVAA<br>CAGACAACAACCGVCCCCC<br>AMACGAGCMCCAACAACC<br>CACAAAAARGRGCCCCCAA<br>SGGGGAAAGRAGGCCACSC<br>CAAAARGARGCGCCAARAA<br>AGGAAARAAAGGGAGCAG<br>CAGAAAAGCCGCAAGGAC<br>AGCCAGMAGAGGGRGCAG<br>ACGCSCCACCTTTTGTAATA<br>AAAGAAGTAAAGCCCCCCC<br>CCCCCCTTTAATAGAAGGG<br>AAGGAGGGGAGGCGGCTG<br>CCGAGGAGCCTCGCCGCAG<br>ATAGAGGCAGAGAGRANG<br>AGAGAGAGNANAGAAGTG<br>GATGAGAGGAGTTGGTGGC<br>CGGCCACCAACTCCGGCGW<br>GCTCGAAGCTCCAGCCACC<br>TCCCTCCCCTCCATWAAGG<br>GGGGGAARGSAATGAKGA<br>AVMAAGNAAGDWAATGGR<br>AAMWTCTTCCCCCTTTTCC<br>CYTTTTTYYTTTTYCTACA<br>WWAAGAGAAGAAVGTDTT	559

	CACMMYYTRGSGRCYMTAYGACGATARKSKKGGTAGCTCGGATGTGGCCTCTTGTTTG	
EtalSat41-506	TTGTTTGACCAAGCCATAGACCAACBBBTCTTATGVCCAATTTVGGTHACGDTSGCTCAATGAGCCTAACYCACACWCCCATGGCSGGCSAAGYSSSTTTTCCATGTCATATGGACACCTAAGAMMAAGGGGGACCCACTTTCCACCTTGGTYAARGGCTTTGGAGGGAAACACCCAAGVTTNCGATGGCAAGCCTCCTCCAGCAGCCATGGCAGGAGGAGGAATNCCATCCACCCTAGCCCACTCCKCTCCTTTCCCTCTTTTTCCACTAGAAAAATTAAAAAATTAAAAAGGASACTCATAGGGAGCTCCCTAGGCATCTCCCMYCCAAADATCTCCCCTCCTAGRGAAMATBGGRGRAGGRAAAGAGCWTAACCCGTAGCTAATAGSATTASCCNAGCNGYCNNGCTCHAMYMMRASWSARASMGAAGAGAGATTTTTGATCTCCCTCCACACTCCACAAAAACTCAAACCAAGGCTCCCACATTGCTCAGTGAGTTCACACA	506
EtalSat62-277	TCTAAATTTGGAAATTTTTCATYRBTTVAATATACTWMMAACATGAATSAAWTAYTTCAAAACAGAGAYCAAAACATAAACGAAAATACAATGATAATTAAAGAACATTTTCTACTCATATTCTTCTTCTTCTTCTTCCTCTTCTTCTTCTTCTTCATCTTCTTCTTCTTCTTCTTGCGGAGGAGTATACCTTTTGTAAGTTGGAAGCGTCGGATAATTAATTGTACTTGGGTCGACATTCAAGCTACAGTTTTGGAAAATTTGATTCATGTTGGAA	277
EtalSat64-311	TTTAGGGGTTTTTACCTACAGGGCACTTAAAAGAGCCCTATTTACCTATTAAGCACCCAAACTATCATATTTACCWRCAAAGCACTCTCCGTTTNCTCCGMACTCTCGTCTCCGT	311

	CAGCCGAGCCGCACGARAA<br>CCCTTNCGNTTCCTNTCTCC<br>TCCTCGTCTCCGTTCGGCGA<br>NNGGCGCGCGCGAGGTCTC<br>CCCTCTCCTCCTCCNACCTN<br>GTTCGGCCACGCMGGCGCG<br>GACSAGANCNAGAGGTGCT<br>TYSCGTSCGCCTCGMCGWA<br>CGGAGACGAGGARGAGAA<br>SCGGGGCTTGCGTGCSSCTC<br>GGC	
EtalSat7-533	GAACTGGACCTCTTTGGGT<br>CCGGGCGACCTAGCCGCCC<br>CACCCTTTCCCTCCTCTTTG<br>TMWWTSWWTMSYWWWW<br>WGYTKRDATTTANCCTAAA<br>CCTAATAAGGGGGGTKTTD<br>TTTGTTTTTKATATTTCAGT<br>TTTGTTTTGTGAGAGTGTTG<br>WTCGTCGTCCGGGGAATCC<br>GATTCCCTACTCTTCTCTCC<br>CGGGTGGTGATCCTGTATC<br>CCGGCGAGGGCGGAGCNTA<br>AAGCCGCCGGCCCCTGAAT<br>TTCCCTAGGCCGATTTTCCA<br>AATAWGGAAAATCGCCCT<br>AGGGAAAATTAGGGGCCG<br>GCGGCTTTAGGGGGGGAAG<br>AAGGGGAATTCGGAGGAG<br>GTAGGGGAAGAAGGGGAR<br>GGCTCCGCCCTCGCCGGGA<br>TACAGRATCACCACCCGGG<br>AGAGAAGGGGATGGAATT<br>GGATTCCCCGGACGAACGA<br>HCADCKCAYAAANTCTCAA<br>AACTGAAATCTGAGAAAHH<br>HTAACVCCCSMWWYKMKR<br>GWTTCGGCGTCAGGGTGTG<br>TCATCTCAACCCCTATTAG<br>AGCCA	533
EtriSat147-118	CTTTCCATCGCGTTATGGAT<br>CGACCCCTTGTCTAGTTGTT<br>TGAAGAATAGGCTGACTTC<br>TACCGGTCACCACCTATTC<br>CTAGATTAGGCATTGGACA<br>CCTTCTACACTACAGGTTG<br>GT	118
EtriSat6-170	CACCCTAACTTTGACCTATT<br>TAGGTCAAGTGGGCCCCAC<br>TTAGGGTTTGGGTCTTGACT<br>AGGCTCAAAGTGTGACCAG<br>ACCCAATGGGTCCCAAACT	170

	TGTCTAGTAGGCTTCTAGT<br>GTATGTGTGAAGGTTTGTG<br>GGAAGTTTGGTCCAATTTG<br>GAGATGTTTGACCAAA	
EtriSat243-199	TCAAATCATCTTTCCTATCC<br>ACCTCCCTTAGTCAGCAAA<br>CTAATAGGTGTAGGAAAAT<br>TTGATGATTTTTCAGGCTAT<br>CGCGCCAAAAGCCTTCATG<br>CCAAGGCCTTCTAGCGCGT<br>ACGCCACTTCATATATCTA<br>AATCAATCGGCCCTCGCGC<br>CGCTAAATCTATCGGCCCT<br>CGAGCCGAAATTTACTTTC<br>AGCATTT	199
EtriSat473-459	AGAGAAGCAGTAGTCGCGA<br>TGAGAAGAAAAAAGTGAG<br>AGGTGTGTTCCACAAAGAA<br>GAGAGAGACGACAGTCGTT<br>GGAATAAGGAATGGGGAG<br>GCAGCTACAAGAAGAAGA<br>CGAATGAGATGGATGGTAG<br>TGGTGAGAAGAATGATGAA<br>GAGGATGAGGGTGATTTTG<br>GTAGCAATGGAAAGAAAA<br>AAGAGGTTGGTGATAGCGA<br>GAAGAATGAGAGAGAAGA<br>TGTTAGCGAGAAGAAAAAG<br>AGGAGAGAGTGTAGTTGTG<br>GTGTAAAGAATATAATGGA<br>TGGAGTACGCTGGGAGAAG<br>GAGGGAGCTGATGACGAG<br>AAGAAGCAGAAGAAGGAG<br>AGGGAGTGGAACGGTGGTT<br>ATTGGTTGGAAGAGGAGGG<br>AGGTAGTGGCCGTCGGAAG<br>GAGGAGGGTGGTGGCAATC<br>GCAAGAAAAAAGAGGAGG<br>TAGATGGTTGCGGTGGCGA<br>GAAGGAAGAGA	459
EtriSat3-405	AAACTAAGGCTGAGTTTGG<br>CCCAGCTTMWYMYRTCAB<br>BCACHBCTHGNNATGYRCT<br>CACAVAGCWGCDCWTTBM<br>AAAAGTAGAGMTAAGCAT<br>ATCTASACAGCAGCTGCAC<br>AGAAAAGCTGGGCCAAACT<br>GAGACTTAGGGTCAGTTTG<br>GCCCAGCTTCGTCTGRCAG<br>CTGGCAGCCGCTTCTTCTGT<br>CAGCTATTTTTTGCATCACT<br>CACACAGCTGTGTGACAGC	405

	TGAGNTTGAGAGAAGCAAGAACAATTGCTGACAGMAGAATCTCCTGACAGCNCANCACAGCCGGGCCAAACTGACCCTAAGTCTGSAYTTGCYTCTGYTTAKTAGTACAGWTKACTTCATCWGAAWCTGATGTYTTCCATTATAATTDTMCTTASAAAMAAGTGDRKTTTCCAAACAC	
EtriSat37-456	TTCACGTGCTATGTATGTAAATTTCCCTTTTCTTTTTCATMTYYTTHACTYMATATATAWWYATAWWTAWCTTKAAAWATWCCTYATTTTAATWTAGGGGTTTTTTCATACATAGCACGCGAAACTTGTGTATTTNCTACATCACGCGGCCTCTCACTGTAGCAGTGACAGCCACAGTGAGAGGCCGCGTAAATACACAAGTTTCGCGTGCTATGTATGTAAAAATCCCTATAAAAAAATRTATAAACACTAAAATAACYYAACTCTAGTAHGTGSKRSKCAYMAATCCAGAGTTACYWCTSATTCWYTCCAGACTGHGDGVCATGTDTGRRKATRRARMRAMMKSMASGCGACTTTGATCGTCTCAAATCTTCCTATGATGCTTTGTTCGCCGTCGTCGGCCCGCTGCCGTCGTCGCCGGAGAAAAGCTGGCAAAATTGGTTGTATCAGGTTAGA	456
EtriSat51-408	AAATATGTGAGCTTRMKCKMGHTVATVCTCADAAGCGGAACAYARACTAGCGTSAWANAYAWTTGRMAAATTTACDAAATTTTAGGGATATTTACATACATACCACCCAATTCTTATGTATTTACATATCTAACACCTAAATTTTAATATTTACATACATACCACCCGATTCTTTTATATCACATCAAAAATACCACTATTGATATTTTTTCAAGTGAAATGCACTTTTTAAGCATAAGAAGGTGGTAWATWAGTAAATATGAAAATTTAGGTGTTAGATATGTAAATTCATATGAATTGGGTGGTATGTATGTAAATTTCCCT	408

	AAATWAWTTAGTATWTAT<br>AYAACAAAAAGATAAGTAT<br>CWAMTTGWMRATGATACK<br>ATTTRCBGAGCHGTGGHBG<br>AMRSGMKTTC	
EtriSat67-399	TCTTTTTACTACTATTTCTT<br>CTTCTTTTACTACTTATTC<br>TTCTTTATACTASTTCDTCT<br>TCTTCTTTATACTACTTCTT<br>MTTCTTCTTTATAMTACTA<br>CTTCTTCTTCTTTATACTAC<br>TTCTTCTTCTANNAANTACT<br>TCTTCTTCTTCTTTATACTA<br>CTTCTTCTTCTTCTTTATAC<br>TACTTCTTTCTTCTTCTTTAT<br>ACTATNTAAATAATAATAA<br>TAATTCTTCTTWATACTACT<br>NTTCTTTTNATAAANATACT<br>NTNTTTCTWTATASAAGAA<br>GAACTAGTNTNAANAANTN<br>NTTCTTTAAAGAAGAAGAA<br>GTAGTTGTTTATACTACTTC<br>TTCTTCTTCTTHATACTACT<br>TCTTCTTCTTATTTATACTH<br>TTCCKCTWCWYCTTTATAT<br>TATT	399
EanoSat45-75	AGGGGTTGGTGGGTTGTTA<br>ATATAGGGAGGAGATGATG<br>GGGAAGGAGAAAGAGAGG<br>GTGACAATGGAGAAGGAG<br>G	75
EanoSat42-144	ATGGTGGGGGAGGTGATTT<br>GTAGTAGTATGGAGGAGGT<br>GGTGAAGGAGATGGGGGA<br>GGAGGAGACTTATAGTGAT<br>ATGGAGGTGGAGGAGAGG<br>GTGATGGTGGAGGTGGGGA<br>TTTATAATGGTATGGTGGA<br>GGAGGTGATGGTG	144
EanoSat145-390	GTGACGAGAAGAAAAAAG<br>TGAGAGGTGAATTCCACGA<br>GGAGGAGAGAGACGGCGG<br>TCGTGGGAATAAGGAATGG<br>GGAGGCAGCTACAAGAAG<br>AAGACGAATGAGGAAGAG<br>GATGAGGGTGGTTTTGGTG<br>GCAATGGCAAGAAGAAAG<br>AGGTTGGTTATAGCGAGAA<br>GAAGGATAAAGAAGATGTT<br>AGAGAGAAGAAAGAGAGG<br>GGAGAGTGTAGTCGTGGTC<br>GGTGGAAAGAATATACAAA	390

	GGAGGGAGCTGATGACGA<br>GAGGAAGCAGAAGAAGGA<br>GATGGAGTGGAACGGTGGT<br>TTTTGGTTGGAAGAGGAGG<br>GAGGTAGTGGCCGCGGGAA<br>GGAGGAGGGTGGTGGCAAT<br>CGCAAGAAAAACGAGGAG<br>GAGAAGCGAAGCAGTGGTC	
EanoSat76-369	GTGCTTTGGTGGCTTGGCT<br>AGYRCDCTBTGTGTGYKTT<br>TTKGKTGGGCTTTTKKGGST<br>CSGGTTTGGKGCTYTTTGGS<br>CTGCCTTGGTGGTGGTGCTT<br>TGGTGGCTGGTTAGTGCAC<br>TTGGTGTGTGCTGTTTGKG<br>KGCTTTTGTGGCTCGGGTTT<br>GGTGCTTTTGGGCTGCCTTT<br>GTGGCTGTTTTGGCTGGTG<br>CACTTGGTGTGCTTTGTTTG<br>TGCACTTGGTGTGTGCCTTT<br>GGTTGGACCTTTGTGGTGC<br>TTGCTGTTTTGCACCCCCCC<br>TTTTCCCTTTTTGCTTTTTTT<br>CTGGGAATGTGGGAAAGTC<br>NTTTTTTTTTGTTTAGAGAG<br>AAAAGGGAAGAAGGGGGG<br>GGCKGYGCYCKYTCT	369
EanoSat23-512	AACCGAGCCAACCAACAAG<br>GCCCVAAVAAVAGGAGCC<br>AAAAGCCAACCAACAGCCV<br>VVACCAAAGVCCAAAGMG<br>AGGMGCCAAAARAGCCAC<br>AAAAARGACCAAMMAAGC<br>CMACCAACAAGGCCCAAR<br>AGGGRGGCAACCARAGMC<br>MAAACCRAGCCCCAAACA<br>GCCCCMMACAAAGGCCCC<br>AACMAGGCCAACCACCGG<br>GCACCCAACAAGCCACAGG<br>AAGGGCTACACCAAGGGA<br>GTAAAAGAGGGCCCAGAA<br>AAGCCGCAGAACCAGCCGG<br>CCAAGCCAGGGGACAGCCG<br>CCCCAAATAACGGAAAAGG<br>AAAAGAAGGACAGCCGCC<br>CTCAATTAATGCAGGAGGA<br>GACCAAGCCACAGCCCCCA<br>ATACAAAGGGGAAAAGCA<br>AAACCGAGGCCANAACAA<br>CAACAACAACAGCAAGAA<br>ACCGCAGAGGAGCCAGCCA<br>CCGAGAAAGAGAAGGAGA	512

	AGAGAGGAGAGGAAGAAG<br>AGCACAGSCCGGCCACCAC<br>CTCCGGCCAGCCCGGC	
EanoSat7-481	GCAGGGAGGAGACMAGCC<br>ACCACAGCCCCCAGTACAA<br>AGGGCAAGAGGAAAAACC<br>GAGNNCAAANAACAACAA<br>AAGCACAACAACAACAAC<br>AGCAAGRAGCCRCAGAGA<br>ACCCACCTTTTTTTCTGGG<br>AATGTGGGAAAGTTTTTNT<br>TTAGAGAGAAAAGGGAAG<br>AAGGGGGGGCTGCTGCTCT<br>TCACCTTCTCCTCCCTTCTT<br>CTTCCTCCCTTCTTCGGCTG<br>CTTGGTTGCCTGGGTGTTGC<br>TKCTGGGAGGCGGGCSGGA<br>GGTGGGGVCCGGCTTGCTC<br>TTCTTCTYCTCCTCTCTTCT<br>CCTCCTCTTCTTCGGCTGCT<br>TGGTTGCCTGGGTGTTGCT<br>GTTGGTGGGSGGGCGGAW<br>GTGGTKGYCGGCTGGTSCY<br>TKTTTTCCCYYCCCCYYTTC<br>TCCTTCTYTCTCSGTGGGTG<br>GSTCTYTCTGSGKTTTTTYT<br>TKGTTGTKTTTSTTTTTTTTS<br>TGSYCKSKKTTKTSTYYYCC<br>T	481
EbarSat63-144	GGAGATGGTGGAGGTGGTG<br>ATTTGTAGTAGTATGGAGG<br>AGGTGGAGAAGGAGATGG<br>TGGAGGAGGAGACTTATAG<br>TGATAGGGAGGTGGAGGA<br>GAAGGAGATGGTGGTGGTG<br>GGGATTTGTAATGGTATGG<br>TGGAGGAGGTGAT	144
EbarSat129-459	CTACAAGAAGAAGACGAAT<br>GAGATGGATGGTAGTGGTG<br>AGAAGAATGATGAAGAGG<br>ATGAGGGTGGTTTTGGTAG<br>CAATGGAAAGAAAAAAGA<br>GGTTGGTGATAGCGAGAAG<br>AAGGAGAGAGAAGATGTT<br>AGTGAGAAGAAAGAGAGG<br>GGAGAGTGTAGTCGTGGTG<br>TAAAGAATATAATGGATGG<br>AGTACGCTGGGAGAAGGA<br>GGGAGCTGATGACGAGAA<br>GAAGCAGAAGAAGGAGAG<br>GGAGTGGAACGGTGGTTTT<br>TGGTTGGAAGAGGAGGGA	459

	GGTAGTGGCCGTCGGAAGG<br>AGGAGGGTGGTGGCAATCG<br>CAAGAAAAAAGAGGAAGT<br>AGATGGTGGCGGTGGCGAG<br>AAGGAAGAGAAGAGAAGT<br>AGTAGTCGTGACGAGAAGA<br>AAAATGTGAGAGGTGTGTT<br>CCACAAAGAGGAGAGAGA<br>CGACATTCGTTGGAATAAG<br>GAATGGGGAGGCAG	
EbarSat20-487	TTCTTCTTCTTTATACTACT<br>TCTTCTTCTTCTTTATACTA<br>CTTCTTCTTCTTTATACTAC<br>TTCTTCTTCTTCTTCTTTATA<br>CTACNTTCTTCTTCTTTATA<br>CTACTTCTTCTTCTTCTTTA<br>TACTACTTCNTTCTTCTTCT<br>TTATACTACTTCTTCTTCTT<br>CNNCTTCTTMTTCTTCTTTA<br>TAATACGAATTATTATTMT<br>TTATACTAATTATNTTCTTC<br>TTSTTTATACTANNNNNTA<br>WAGAANNAGAAGTTGTTCT<br>TCTTGTTTATASTACNNTTC<br>TTCTTNNNNNTNNTTCNTCT<br>TNNTATATNCTTCTTNTTTN<br>AAAGAAGAAGAAGAAGTA<br>GTATAAAGAAGAAGANNN<br>NNNNNNNNNNNAAGAAGW<br>AGTATWAWRAAGAAGAAG<br>AAGAAGTAGTATAAAGAA<br>GAAGAACAAGAAGTAGTAT<br>AAAGAAGAAGAAGAAGTA<br>GTATAAAGAAGAAGAAGA<br>AGTAGTATAAAGAAGAACA<br>AGAAG	487
EbicSat47-75	TGTCACCCTCTCTTTCTCCT<br>TCCCCATCATCTCCTCCCTA<br>TATTAACAACCCACCAACC<br>CCTCCTCCTTCTCCAT	75
EbicSat45-96	TAGGGAGGAGGAGGAGAG<br>GGTGATGGTGGAGGAGGTG<br>ATTTATAGTGGTATGGAGG<br>AGGTGGAGATGGAGATGGT<br>GGAGGAGGAGACTTATAGT<br>GA	96
EbicSat51-170	CGAACATCTCCAAATTGGA<br>CCAAACTTCTCACAAACCT<br>TCACACATACACTAGAAGC<br>CTACTTGACAAGTTTGGGA<br>CCCATTGGGTCCGGTCACA<br>TTTTAAGTTTAGTCAAGAC	170

	CCAAACCCTAAGTGGGGCC<br>AATTTGACCTAAATAGGTA<br>AAAGTTAAGGTGTTTTTT	
EbicSat177-432	CGCTGGCAGAAGGAGGGA<br>GCTGATGACGAGAGGAAGC<br>AGAAGAAGGAGATGGAGT<br>GGAACGGTGGTTTTTGGTT<br>GGAAGAGGAGGGAGGTAG<br>TGGCCGCGGGAAGGAGGA<br>GGGTGGTGGCAATCGCAAG<br>AAAAAAGAGGAGGTACGC<br>GGTGGCGGTGGAGAGAAG<br>GAAGAGAAGAGAAGCAGT<br>GGTCGTGACGAGAAGAAA<br>AAAGTGAGAGGTGAGTTCT<br>ACGAGGAGGAGAGAGACG<br>GCGGTCGTGGGAATAAGGA<br>ATGGGGAGGTAGCTACAAG<br>AAGAAGACGAATGAGGAA<br>GAGGATGAGGGTGGTTTTG<br>GTGGCAATGGCAAGAAGA<br>AAGAGGTTGGTTATAGCGA<br>GAAGAAGGATAAAGAAGA<br>TGTTAGCGAGAAGAAAGAG<br>AGGGGAGAGTGTAGTCGTG<br>GTGTAAAGAATATAATGGA<br>TGGAGTA	432
EignSat47-96	GAGGAGAAGGTGATGGTG<br>GAGGTGGTGATTTGTAGTG<br>GTATGGAGGAGGAGGTGA<br>GGGAGATGGTGGAGGTGG<br>AGACTTATAGAGATAGGGA<br>GGAG	96
EignSat100-170	GGTTTGTGGGAAGTTTGGT<br>CCAATTTGGAGATGTTTGA<br>CCAAACACCCAAACTTTAA<br>CCTATTTAGGTCAAGTGGG<br>CCCCACTTAGGGTTTGGGT<br>CTTAACTTGGCTCAAAGTG<br>TGACCGGACCCAATGGGTC<br>CCAAACTTCTCTAGTAGGC<br>TTCTAGTGTATGTGTGAA	170
EignSat1-683	GAGTGAAAGAGAGAGAGA<br>GAGAGGAGGGGGAGRSAKS<br>KRGWRAKASWSWRAKARK<br>MAKAKASARAKSRAAASM<br>RRRWSMRMAHBVDHDDGC<br>VATADCTAGAGADVAAGA<br>GAGVGAGAGAGAGAGAGA<br>GAGAAAGAGDGAGNNAKM<br>TAGAGAGAGASNGAGAGA<br>GAGAGAGGGAGGGGAGGA	683

	AGAGAGAGGGGAAGGGAG<br>GGGGAGAGAGAGAGARAG<br>AGAGGGAGAGGAGGAGAG<br>AGAGCGCTCTCTCTCTCTCT<br>CTCTCTCTCTCTCTCTCTCT<br>CTCTCTCTCTGAGAGAGAG<br>AGAGAGAGAGAGCGAGAG<br>AGAGAGAGAGAGAGAGAA<br>GAGAGAGAGAGAGAGAGA<br>GAGAGAGAGAGAGAGAGA<br>GAGCCTCTCTCTCTCTCTCT<br>CTCTCTCTCTCTCTCTCTCT<br>CTCTCTCTCTCTCTCTCTCT<br>CTCTCTCTCTCTCTCTCTCT<br>CTCTCGCTCTATCTAGAGA<br>GAGAGGGAGAGAGAGAGR<br>GAGARAGAGVGAGAAAGR<br>GAGGGNGAGAGARAGAGA<br>GAGAGAGAGAGAGGGAGA<br>GAGAARGRGRGAGAGAGA<br>GMTCANKASTGASAGMTAG<br>NGTGACAGANAGASCGNCN<br>NNCCTCNGVVRRRARRKRR<br>RRRRRRRKWRKRWRWWGGR<br>RKRRRRGRRGGGAKRRRRR<br>RRRRRRKRRRRRRRRRGGG<br>AGAGAGCGCTGTGAG	
ElauSat44-75	CCAACCCCTCCTCCTTCCCC<br>ATTGTCACCCTCTCTTTCTC<br>CTTCCCCATCATCTCCTCCC<br>TACATTAACAACCCA	75
ElauSat66-159	CCACCTCCTCCTCCCTACAT<br>TTACAACCCACCACCTCCA<br>CCTTCTCCTTCTCCACCGCC<br>TCCTCCTCACATTTACAACT<br>CCCCACCTCCTCCTCCTCCT<br>TCTCCATCACCTCCTCCTCC<br>CTATATTTACAAGTCACCA<br>CCTCCACCTTCTCCTTCTCC<br>A	159
ElauSat401-110	GGGTCCTTCTTCATCAAGTC<br>CTAGCATATAGGCTTGATG<br>ATTCATCAGGAACTCTTCA<br>TACCCTACTATGAAGAACC<br>CTGACTCTTCCCAGTGCTTG<br>GCGTCCCGGGCCT	110
ElauSat13-149	CTCTAGGAGATGGGAAATT<br>CGGCTAAGCCAAGGGAGG<br>ATTGATTGGATGATTTCCG<br>ATTGGAGAGAGAGGAGGA<br>GAGCTAGGGCCGGCCTTGA<br>CTTCCCCTCTTTCCAAAACC	149

	GCCCCACCCAATCAAGGAG<br>AGGGGAATTTCGGCTAG	
ElauSat36-240	TCTCTATAAGTCTCCTCCTC<br>CCCCATCTCCTTCACCACCT<br>CCTCCATACTACTACAAAT<br>CACCTCCCCCACCATCACC<br>ATCACCTCCTCCACCATAC<br>CATTACCAATCCCCACCTC<br>CACCATCACCCTCTCCTCCT<br>CCTCCCTACCACTACCAAT<br>CACCACCTCCACCATCTCC<br>ATCTCCTCCTCCACCATACC<br>ATTATAAGTCCCCACCACC<br>ACCATCACCTTCTCCTCCAC<br>CTCCCTA	240
ElauSat123-645	ACGAGAAAGAGAGAGAGA<br>GAGGGAGGTCTCTCGGCTT<br>TGCTGTGAGCGTCGAACAG<br>AAGGGAGACGAGAGCGGT<br>CGGCCTCCTGTGTGCGTCG<br>AACAGGAGAAGAGAGGGA<br>GAGAGGCGCCTTTGCGACT<br>CTTGCTGGCGCTCGTTCGA<br>ACAGAGAAGAGGGAGACG<br>GAGGTGCGGCCTTCCCTGT<br>GCGTACGTGCGTGAGTCGA<br>AGAAGAAGGGGGGAGGTC<br>TGCTGCGGGTTTCTGCGTG<br>CGAAGAGCAGAGAAGGGA<br>GAGCGGGAAATGCTCGCGT<br>GAATTAAGCCGAAATTTTC<br>ATCCGTACGCGCTAGAGAA<br>AGGCGAATACGGGCCTTCA<br>GGTACCTGTATGAAAGAGA<br>ATACACTTCTATATTTTTTT<br>CCTTTTTCATCTTCTTTCCC<br>CTCTTCTTCTCTTCCTCCTC<br>CTTCTTCCCTCCAAACTTCT<br>CCTTCACACATAAGTCCAC<br>ATTCACATTCATACATGTAT<br>AAATAATACAATAATAACA<br>ATAATAATGCTATTAATAC<br>TACTATTGTTATTATTATTA<br>TAGAAGAACAGGGAAACTT<br>TTCTCTGTTAATAGAGAAA<br>TTGTTTCTCTGTCGAGCAGT<br>AACCGATCACCTGCAGGTG<br>GAGCGGGTCGGTTCGGGTT<br>GTGCTCGGCTGAAGGAG	645
EoxySat37-327	TGTTCTCAAGTCGTGCGCA<br>AAAGCTAGCGGAGGTCCTG<br>TTCGGAGCAAATTTCTTGT	327

	ATTCTTCCACTAAAAAGG<br>TCCTCCATGAATTTTCTTCA<br>TTTTCGGAAGAACCTTAAA<br>GGTGTTAGCCCATGAAAAG<br>TGCGCTCACAGACAAAAGT<br>TCTACGTCTTCACGTTTTGC<br>GCGCAGATGCTTGCGCAAC<br>ATTTCGCAAAACCACATTG<br>AATCATATTTCATGTATTTT<br>TCACTAAAAATTGATGGGC<br>CATAAGTCTTTTGAACTTTT<br>GTATGATTCTCAAAGGTGT<br>TAAGTCATGAGACGTTGGC<br>TCCGGACACAAAAAATCTA	
EoxySat176-150	ATTATCACCCTCTCTTTCTC<br>CTTCCCCATCATCTCCTCCC<br>TACATTAACAAACCACCAA<br>CCCCTCCTCCTTCTCCATTA<br>TCACCCTCTCTTTCTCCTTC<br>CCCATCATCTCCTCCCTACA<br>TTAACAACCCACCAACCCC<br>TCCTCCTTCTCC	150
EoxySat252-438	ACTCACCTCTCACTTTTTTC<br>TTCTCGTCACGACCACTGCT<br>TCTCCTCTCTTCCTTCTTGC<br>CACCGCCACCGCCTACCTC<br>CTCTTTTTTCTTGCGATTGC<br>CACCACCCTCCTCCTTCCCG<br>CGGCCACTACCTCCCTCCTC<br>TTCCAACCAAAAACCACCG<br>TTCCACTCCATCTCCTTCTT<br>CTGCTTCCTCTCGTCATCAG<br>CTCCCTCCTTCTCCCAGCCT<br>GCTCCCTCCTTTGTATATTC<br>TTTCCACCGGCCACGACTA<br>CACTCTCCCCTCTCTTTCTT<br>CTCGCTAACATCTTCTTTAT<br>CCTTCTTCTCGCTATCACCA<br>ACCTCTTTCTTCTTGCCATT<br>GCCACCAAAACCACCCTCA<br>TCCTCTTCCTCATTCTTCTT<br>CTTCTTGTAGTTGCCTCCCC<br>ATTCCTTATTCCCATGACCG<br>CCGTCTCTCTCCTCCTCGTG<br>GA	438
EphySat91-144	CACTATAAGTCTCCTCCTCC<br>ACCATCTCCTTCACCACCTC<br>CTCCATACTACTACAAATC<br>ACCTCCCCCACCATCACCA<br>TCACCTCCTCCACCATACC<br>ATTACAAATCCCCACCTCC	144

	ACCATCACCCTCTCCTCCTC<br>CTCCCTAT	
EphySat151-105	TAATATATGGAGGAGGAGG<br>AGATGGAGAAGGAGCAGG<br>AGGAGGTGGGGAGTTGTAA<br>ATTTGAGGAGGAGGCGGTG<br>GAGAAGGAGAAGGTGGAG<br>GTGGTGACTTGT	105
EphySat140-314	TCCCTCSCTCTCTNNNNNCY<br>CTCCCTCTCTCTCTCCCTCC<br>CNAGAGANAGAGAGGNNG<br>GGAGGGAGAGAGAGAGAT<br>AGAGAGGGAGAGGAGAGT<br>GAGAGNGGAGAGAGAGAG<br>ATAGAGAGTCAACCAAATT<br>CCACCAGGAGGGAGGGAG<br>GGAGAGGGAGGGAGAGAG<br>AGGGAGAGGAGAGAGAGA<br>GGNNNGNNNANNNNNGGN<br>NANGAGAGNGAGAGNNNN<br>NNTNNCTCTCTCTCTCTCTC<br>CCTCCCTCTCACTCTCCTCT<br>TCCTCTCTATCTCTCTCTCT<br>CCCTCCCTCCTCTCCTCTAT<br>CTCTCTCCCTCTC	314
EphySat100-323	AAGAGAGAGRGRGRGAGA<br>RAGAGAGGGAGAGAGAGM<br>GAGAGGTGAGAGAGATAG<br>AGAGAGAGAGCGGAGAGA<br>GAGAAAGAGAGATAGAGA<br>AGAGGGAGGGATGGAGAG<br>AGAGAGANAGAGAGAGAG<br>AGAGAANNNNNNCTNTNCC<br>TCCCTCTTNNNAGAGGAGA<br>NGAGAGNGAGAGGNAGAR<br>AGAGGGAATNANNNNNNN<br>NTCTCTCTCTCTCTCTCTCT<br>CTCTCCATCCCTCCCTCTTC<br>TCTATCTCTCTTTCTCTATC<br>TCCACTCTCTCTCTATCTCT<br>CTCTCCCWCCTCTCGCTCTC<br>CTTCTCTCTCTCHCWSMSW<br>CCCT	323
ErouSat160-111	AGGAGTCTAATCCGGTCTA<br>ACCAAGGCTAATGGAGGTC<br>TTATAGGAGAGAGGTGGTC<br>CGGTCCTATGAGAGTCCTA<br>AAGGAGATATGCGGTTGGT<br>TGGAGGGTGACTATCT	111
ErouSat267-456	TGAGGAAGAGGATGAGGA<br>GGATTTTGGTAGCAATGGA<br>AAAAAAAAAGAGGTTGGT	456

	GATAACGAGAAGAAGGGG<br>AGAGATGTTAGCGAGAAGA<br>AAGAGTGGGGAGAGTGTA<br>GTCGTGGTGTAAAGAATAT<br>AATGGATAGAGTACGCTGG<br>GAGGAGGAGGGAGCTGAT<br>GACGAGAAGAAGCAGAAG<br>AAGGAGAGGGAGTGGAAC<br>GGTGGTTTTGGGTTGGAAG<br>AGGAGGGAGGTGGTGGCC<br>GCGGGATGGAGGAGGGTG<br>GTGGCAATCGCAAGAAAAA<br>AGAGGAGGTACGCGGTGGC<br>GGTGGAGAGAAGGAAGAG<br>AAGAGAAACAGTGGTCGCG<br>ATGAGAAGAAAAAAGTGA<br>GAGGTGTGTTCCACAAAGA<br>GGAGAGAGACGGCAGTCGT<br>GGAAATAAGGAATGGGGA<br>GGCAGCTACAAGAAGAAG<br>ACGAATGAGATGGAGGGC<br>AGTGGTGAGAAGAA	

Legend: The first column indicates the satellite DNA name for each species, the second column contains the complete nucleotide sequence, and the third column shows the sequence length in base pairs (bp).
