# Supplementary figures and images for: The Repeatome in the Mega-Genus Epidendrum L. (Epidendroideae, Orchidaceae): An In Silico Comparative Analysis
Source: Genes (Basel). 2026 Jan 30;17(2):161. doi: 10.3390/genes17020161 (PMC12940908; doi:10.3390/genes17020161)

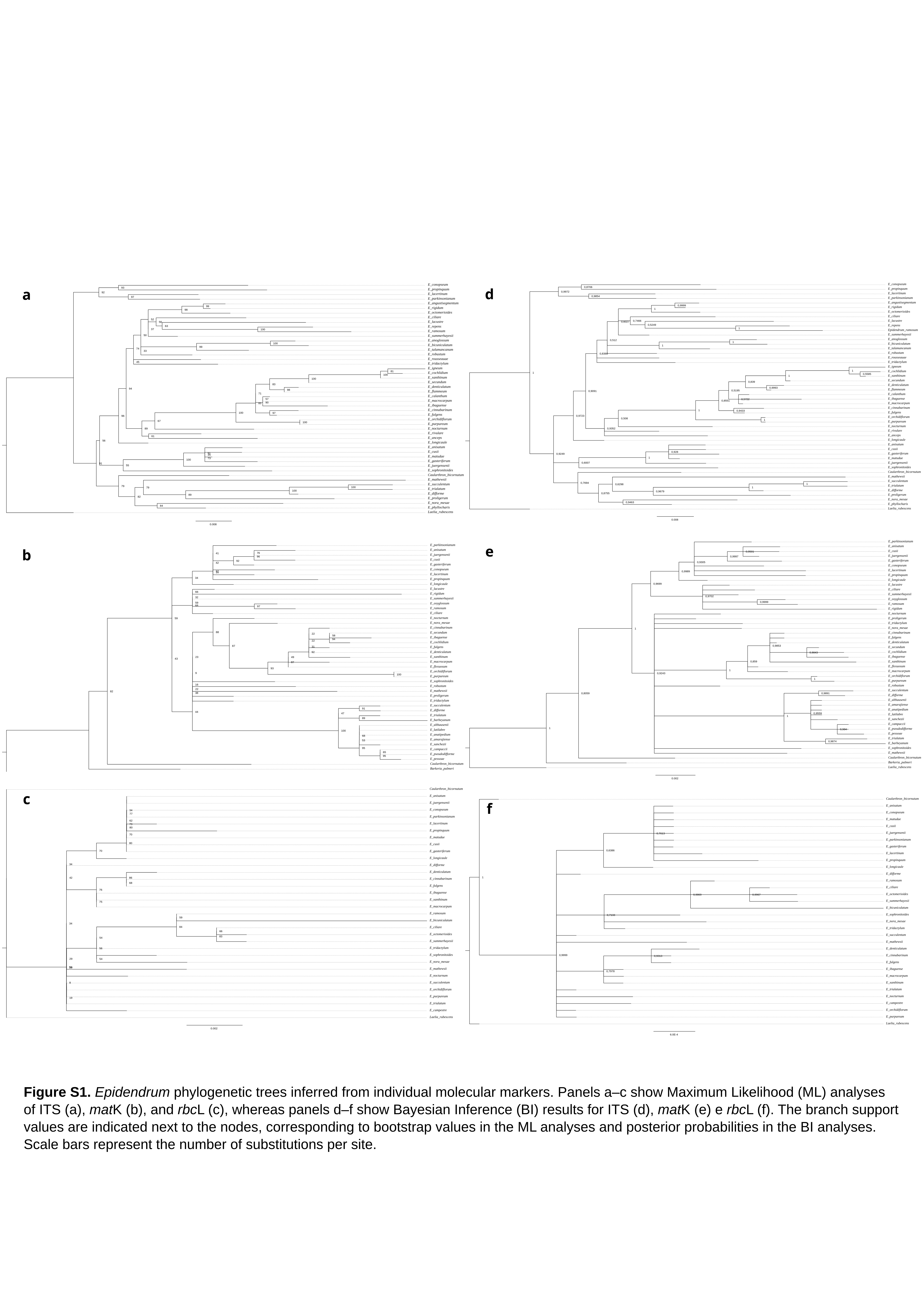

Supplement: Supplementary file 1 [file genes-17-00161-s001.zip › Figure S1.png]

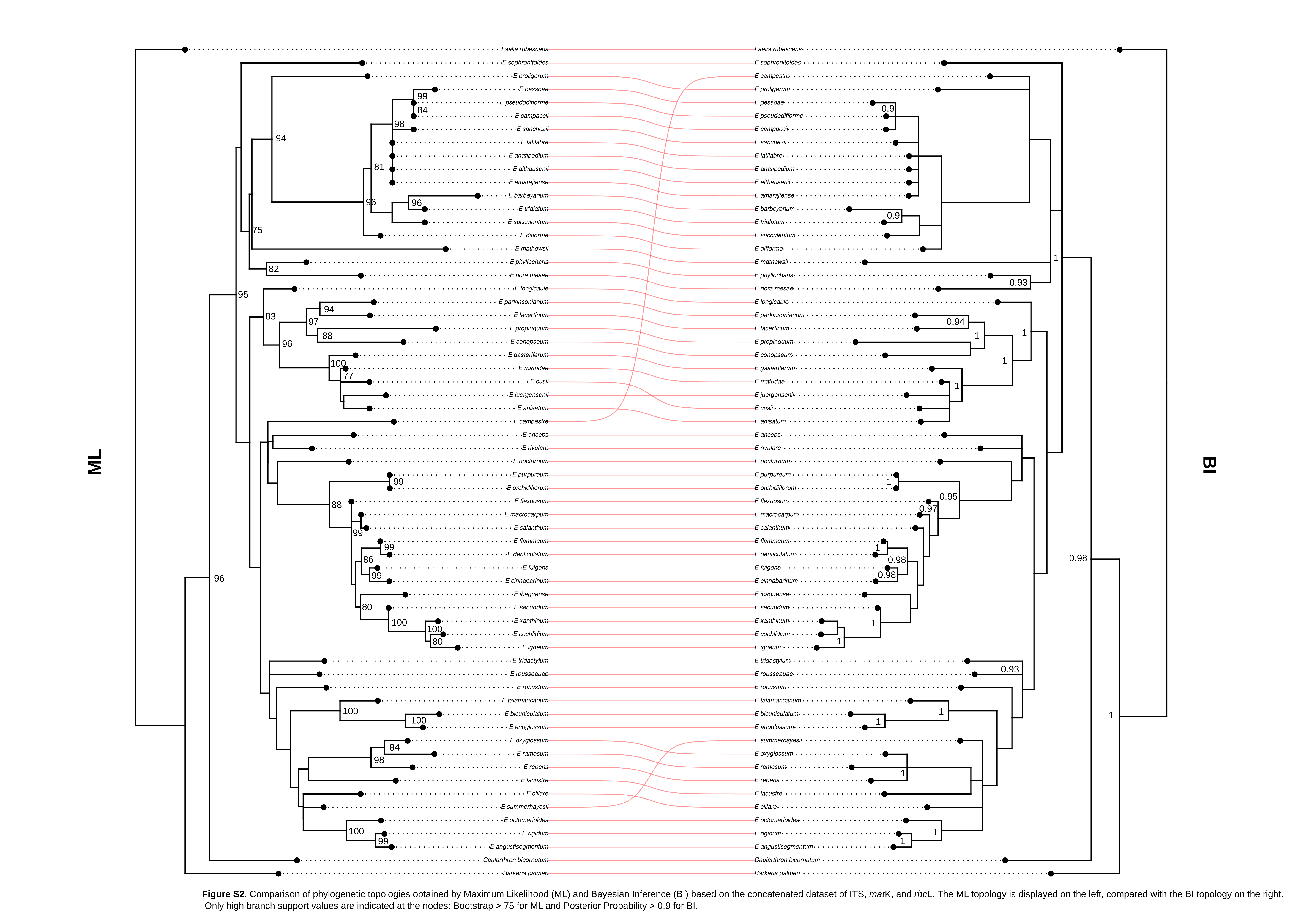

Supplement: Supplementary file 1 [file genes-17-00161-s001.zip › Figure S2.png]

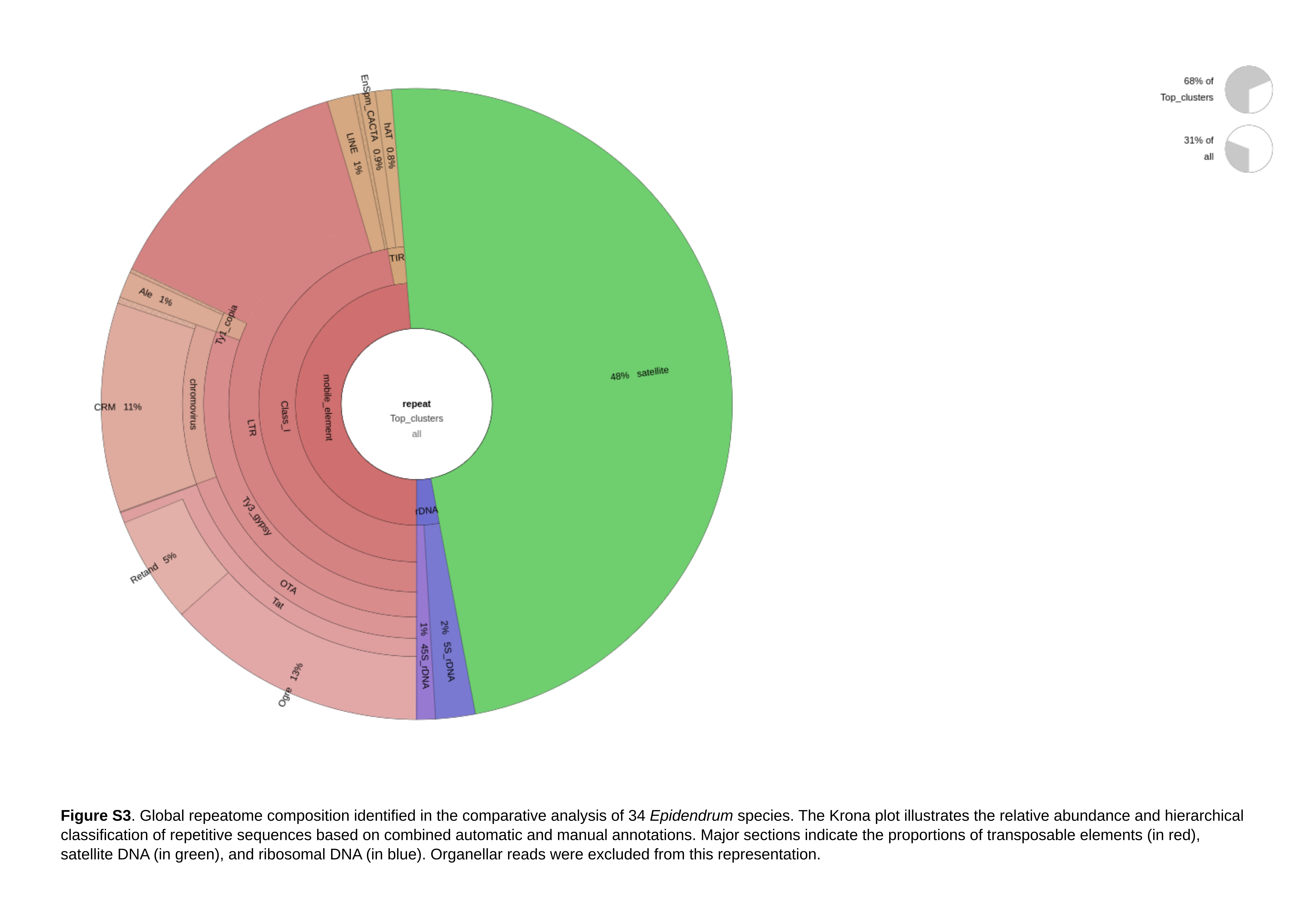

Supplement: Supplementary file 1 [file genes-17-00161-s001.zip › Figure S3.png]
